# Identification of mobile genetic elements with geNomad

**DOI:** 10.1038/s41587-023-01953-y

**Published:** 2023-09-21

**Authors:** Antonio Pedro Camargo, Simon Roux, Frederik Schulz, Michal Babinski, Yan Xu, Bin Hu, Patrick S. G. Chain, Stephen Nayfach, Nikos C. Kyrpides

**Affiliations:** 1 DOE Joint Genome Institute, Lawrence Berkeley National Laboratory, Berkeley, CA USA; 2 Bioscience Division, Los Alamos National Laboratory, Los Alamos, NM USA

**Keywords:** Machine learning, Software, Virology, Environmental microbiology

## Abstract

Identifying and characterizing mobile genetic elements in sequencing data is essential for understanding their diversity, ecology, biotechnological applications and impact on public health. Here we introduce geNomad, a classification and annotation framework that combines information from gene content and a deep neural network to identify sequences of plasmids and viruses. geNomad uses a dataset of more than 200,000 marker protein profiles to provide functional gene annotation and taxonomic assignment of viral genomes. Using a conditional random field model, geNomad also detects proviruses integrated into host genomes with high precision. In benchmarks, geNomad achieved high classification performance for diverse plasmids and viruses (Matthews correlation coefficient of 77.8% and 95.3%, respectively), substantially outperforming other tools. Leveraging geNomad’s speed and scalability, we processed over 2.7 trillion base pairs of sequencing data, leading to the discovery of millions of viruses and plasmids that are available through the IMG/VR and IMG/PR databases. geNomad is available at https://portal.nersc.gov/genomad.

## Abstract (web summary)

geNomad identifies mobile genetic elements in sequencing data.

## Main

Mobile genetic elements (MGEs) are selfish genetic entities that, unlike cellular organisms, are unable to self-replicate and, instead, rely on host cells and cellular machinery to propagate. MGEs are associated with all domains of life and encompass elements with various replication and mobility strategies, such as plasmids and viruses. These elements are ubiquitous in nature and are found across virtually all of Earth’s ecosystems^1,2^. Due to their mobility, plasmids and viruses can serve as key drivers of horizontal gene transfer, a process in which cells acquire genetic information from a mobile gene pool rather than through vertical descent^3,4^. As a result, they play a role in driving fast evolutionary and ecological innovation, greatly impacting the dynamics of all biological communities.

With the increased availability of metagenomic sequencing data from diverse ecosystems, it became possible to study the diversity and distribution of MGEs on a global scale. In recent years, numerous studies have harnessed these data to uncover an unprecedented diversity of viral genomes, greatly expanding understanding of their genetic diversity, distribution, function and evolution. Plasmids, on the other hand, have been mostly overlooked in metagenomic surveys, and most known sequences are derived from clinical isolates and model species, highlighting the need for further research to understand the factors underlying their spread and evolution in natural environments.

Computational identification of plasmids and viruses from sequence data relies on the use of sequence classification models, which can be broadly categorized into two types: alignment-free models and gene-based models. Alignment-free models perform classification directly from nucleotide sequences and employ deep learning architectures, such as recurrent neural networks or convolutional neural networks, to learn discriminative sequence motifs that are informative for classification^5–7^. In contrast, gene-based classification methods perform database searches and alignments to identify marker proteins that are indicative of the underlying identity of the sequence^8^. Both alignment-free and gene-based approaches have been used successfully for plasmid and virus identification. However, most available tools are incapable of simultaneously identifying both classes of MGEs, and currently, there is no algorithm that combines the strengths of alignment-free and gene-based models within a single framework.

Here we introduce geNomad, a tool for concurrent identification and annotation of both plasmids and viruses in sequencing data. We demonstrate that geNomad’s classification framework, which uses a hybrid approach that combines alignment-free and gene-based models, substantially outperforms other plasmid and virus identification tools. Applying geNomad to metagenomes and metatranscriptomes revealed numerous RNA and giant virus sequences that were missed by large-scale surveys, expanding phylogenetic diversity of giant viruses. Additionally, we show that geNomad is computationally efficient and scalable, making it suitable for use in large-scale surveys, such as identification of potential virus and plasmids sequences, across all public sequencing data in the Integrated Microbial Genomes & Microbiomes (IMG/M) database^9^.

## Results and Discussion

### The geNomad framework for classification and annotation

**Fig. 1:**
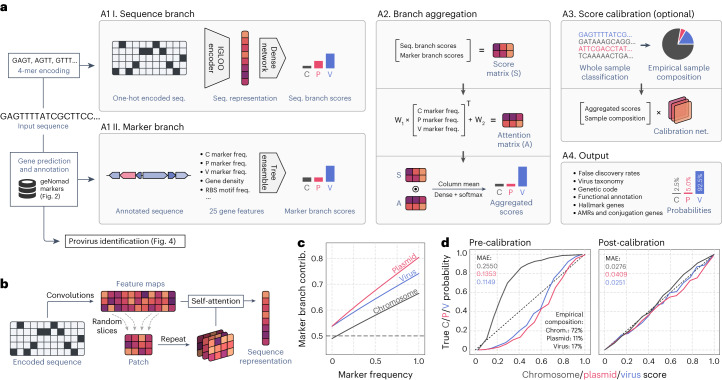
A hybrid framework for identifying and annotating plasmids and viruses. **a**, geNomad processes user-provided nucleotide sequences through two branches. In the sequence branch, the inputs are one-hot encoded fed to an IGLOO neural network, which scores inputs based on the detection of non-local sequence motifs (A1 I). In the marker branch, proteins encoded by the input sequences are annotated using markers that are specific to chromosomes, plasmids or viruses (A1 II). A set of numerical features is then extracted from the annotated proteins and fed to a tree ensemble model, which scores the inputs based on their marker content. Next, the scores provided by both branches are aggregated by weighing the contribution of each branch based on the frequency of markers in the sequence (A2). Aggregated scores can then be calibrated to approximate probabilities in a process that leverages the sample composition inferred from the classification of sequences from the same batch (A3). Lastly, classification results are summarized and presented together with additional data, such as virus taxonomy, gene function and the inferred genetic code (A4). **b**, The sequence branch is based on the IGLOO architecture, which uses convolutions to produce a feature map from a one-hot encoded input. Patches encoding non-local relationships within the sequence are then generated by slicing the feature map. Lastly, these patches are used as an attention matrix to produce a sequence representation from the feature map. **c**, The relative contribution of the marker branch (*y* axis, quantified using SHAP) increases as the marker frequency (fraction of genes assigned to a marker) in the sequence increases. **d**, Calibration curves of pre-calibration (left) and post-calibration (right) scores, showing that sample composition can be used to map classification scores to actual probabilities. The *x* axis represents scores averaged across multiple bins; the *y* axis represents the fraction of positives in each bin; the 45° dashed line represents a perfect calibration scenario. freq., frequency; MAE, mean absolute error of the scores relative to the true probabilities.

geNomad employs a hybrid approach to plasmid and virus identification that combines an alignment-free classifier (sequence branch) and a gene-based classifier (marker branch) to improve classification performance by capitalizing on the strengths of each classifier. geNomad’s framework consists of five stages (Fig. 1a): (1) alignment-free classification in the sequence branch; (2) sequence annotation and gene-based classification in the marker branch; (3) aggregation of the branch scores; (4) score calibration; and (5) output generation.

**Extended Data Fig. 1:**
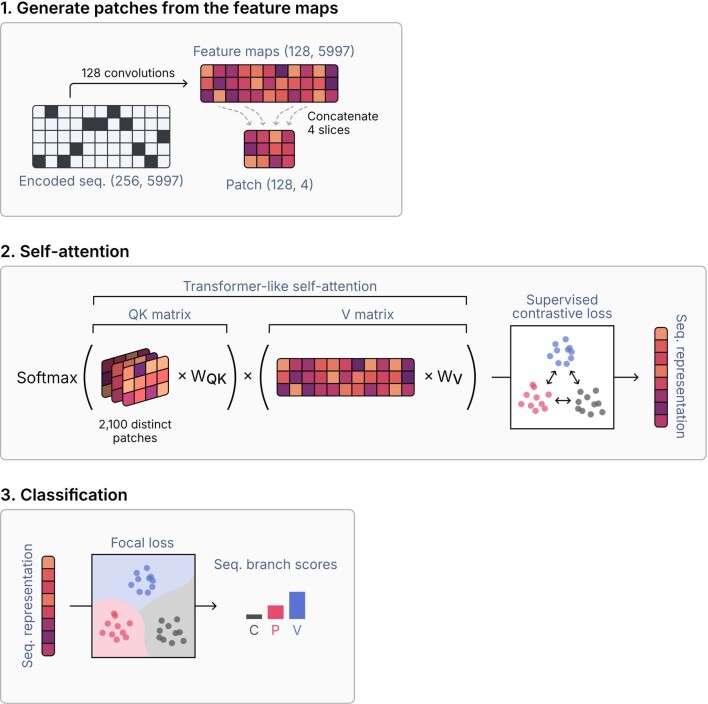
Global sequence representations generated by the IGLOO encoder are used for sequence classification. (**a**) The IGLOO encoder applies 128 independent convolutions to the one-hot-encoded sequence to create a feature map, from which four random slices are taken and concatenated to generate patches that encode long-distance relationships within the sequence. (**b**) A total of 2,100 patches are used to weight different parts of the feature map in a transformer-like self-attention mechanism that results in a high-dimensional sequence representation. The encoder was trained using a supervised contrastive loss function, which optimizes the separation of the three classes (chromosome, plasmid, and virus) in the embedding space. (**c**) To classify sequences, the sequence representations generated by the IGLOO encoder are fed to a dense neural network trained with focal loss to account for class imbalance.

To identify sequences of plasmids and viruses in an alignment-free manner, geNomad’s sequence branch uses a neural network model that can classify the sequences from their nucleotide makeup alone (Fig. 1a, box A1 I). To process input sequences, geNomad employs an encoder based on the IGLOO architecture^10^, which is able to extract patterns that are useful for classification from the nucleotide sequences and encode them into an embedding space (Fig. 1b and Extended Data Fig. 1). This architecture has demonstrated superior performance compared to traditional alternatives (such as recurrent and convolutional neural networks) when applied to sequence data, as it gathers information from non-local relationships across the sequence to create a global representation^10,11^.

**Fig. 2:**
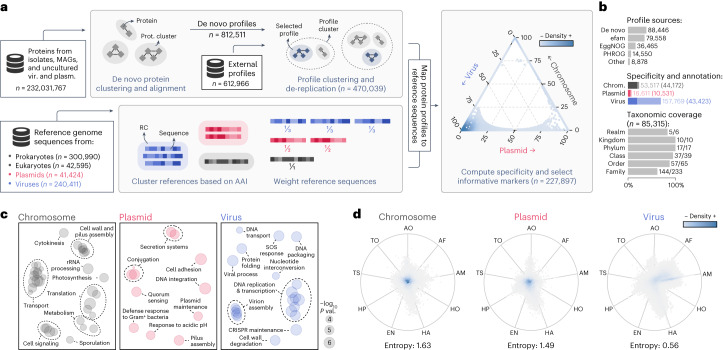
Generating of a dataset of protein profiles with abundant metadata for sequence classification and protein annotation. **a**, Protein sequences from genomes and metagenomes were clustered and aligned to produce de novo protein profiles. De novo profiles and profiles obtained from public databases were then clustered, and cluster representatives were selected to reduce redundancy. In parallel, reference chromosome, plasmid and virus sequences were clustered into RCs. Sequences were then weighed in such a way that the sum of the weights within each RC was constant. Representative protein profiles were mapped to reference sequences, and chromosome-, plasmid- and virus-specificity metrics were computed for each profile based on the weighed number of hits to sequences of each class. Markers that were highly specific to one of the three classes were then selected. The position of each selected marker (circles) in the ternary plot is determined by its specificity, and the colors represent the marker density in a region. **b**, Bar plots showing: the sources of the selected profiles (upper plot); the total number of markers (light shades) and the number of functionally annotated markers (dark shades) for each class (middle plot); and the fraction of ICTV taxa covered by the taxonomically informative markers at each rank. **c**, Multidimensional scaling of semantic similarities of the GO terms enriched in chromosome (left), plasmid (center) and virus (right) markers. Labels of related terms were aggregated for clarity. Semantic similarities were computed with REVIGO. **d**, RadViz visualizations of the relative frequencies of geNomad markers across distinct ecosystems. Each marker is represented by a circle, and the colors depict the marker density within a region. The position of the markers in the plot is determined by their frequency in each environment. Markers close to the center of the plot were found in similar frequencies across all ecosystems. Median entropies of the ecosystem distributions are shown below the plots. AF, aquatic (freshwater); AM, aquatic (marine); AO, aquatic (other); EN, engineered; HA, host-associated (animals); HO, host-associated (other); HP, host-associated (plants); TO, terrestrial (other); TS, terrestrial (soil).

To classify sequences based on their gene content, geNomad’s marker branch predicts and annotates the proteins encoded by input sequences using a set of custom markers (Fig. 1a, box A1 II). To predict proteins, geNomad uses a modified version of the Prodigal^12^ software called prodigal-gv, which we developed to allow automatic detection of recoded TAG stop codons (common in *Crassvirales* phages^13^) and annotation of TATATA motifs that are frequently found upstream of coding sequences of *Nucleocytoviricota* viruses^14^. Predicted proteins are then queried against a set of 227,897 protein profiles—specific to chromosomes, plasmids or viruses (Fig. 2)—using MMseqs2 (ref. ^15^) protein profile search. Next, geNomad computes a total of 25 numeric genomic features that summarize the sequence structure (for example, gene density and strand switch rate), RBS motifs (for example, TATATA motif frequency) and marker content (for example, frequency of chromosome, plasmid and virus markers) of the input sequences (Supplementary Note 1 and Supplementary Table 1). These features are then fed to a tree ensemble classification model, which outputs the confidence scores for each class.

From the outputs produced by the sequence and marker branches, geNomad generates an aggregated classification that leverages the strengths of each approach. This is achieved through an attention mechanism that consists of a linear model that weighs the branches based on the frequency of chromosome, plasmid and virus markers in the input sequence (Fig. 1a, box A2). The attention mechanism works in such a way that the contribution of the marker branch goes higher as the fraction of genes that are assigned to markers increases (Fig. 1c). This allows geNomad to take advantage of both marker-based and alignment-free classification approaches in a principled manner.

**Extended Data Fig. 2:**
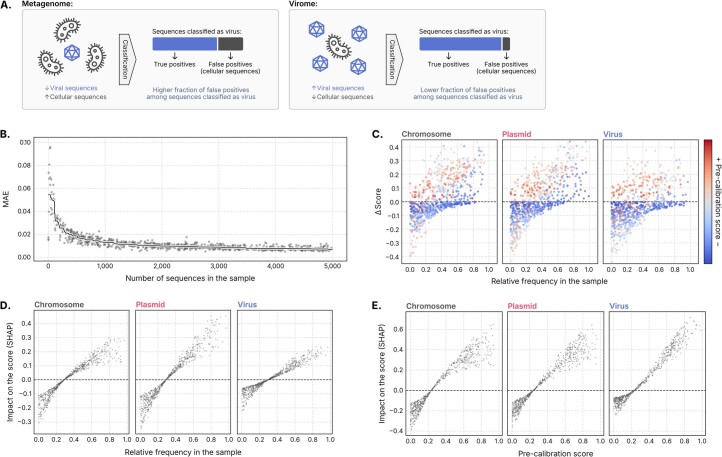
Sample composition can be leveraged to calibrate classification scores to approximate probabilities. (**a**) The false positive rates of a set of classifications depend on the sample’s underlying composition. In typical metagenomes, where cellular sequences outnumber viral sequences, the fraction of false positives within scaffolds classified as viral is higher than in a virome. (**b**) The mean absolute error (MAE) of the score calibration model (*y*-axis) is highly dependent on the number of sequences in the sample (*x*-axis), as larger samples will result in more accurate estimates of the underlying sample composition. (**c**) The calibration model tends to increase the scores of a given class when it is abundant in the sample and reduce the scores when the class is rare. (**d**) The relative frequency of a given class in the sample (*x*-axis) contributes positively to the model output (*y*-axis, quantified using SHAP) when that class is abundant in the sample and negatively when the class is rare. (**e**) The pre-calibration score of a given class in the sample (*x*-axis) contributes positively to the model output (*y*-axis, quantified using SHAP) when the initial score is high and negatively when the initial score is low.

During inference, a classification model assigns a score to each prediction, indicating the degree of confidence in that prediction, with higher values representing more confident predictions. However, these scores do not reflect the true probabilities of the predictions being correct, as classification models will exhibit varying false discovery rates (FDRs) when classifying samples with distinct underlying composition (Supplementary Note 2 and Extended Data Fig. 2). To address this, we devised an optional calibration mechanism in geNomad that leverages sample composition data to approximate the true underlying probabilities. (Fig. 1a, box A3, and Fig. 1d). The calibrated scores produced by geNomad offer users two benefits: (1) estimated probabilities can be used to compute FDRs, allowing users to make more informed decisions (for example, setting a threshold to achieve a desired proportion of false positives); and (2) improved classification performance by adjusting the assigned labels of some sequences after calibrating scores (for more details, see ‘geNomad accurately identifies plasmids and viruses’ section).

**Extended Data Fig. 3:**
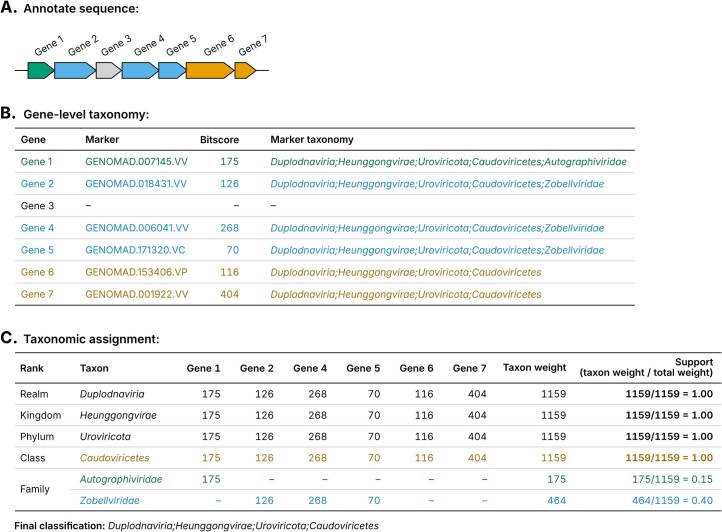
Assigning viral taxa using geNomad’s markers. (**a**) To assign viral sequences to specific taxa, geNomad utilizes a best-hit approach to initially assign the genes encoded by these sequences to markers. (**b**) Each gene is subsequently classified based on the taxonomic lineage of the assigned marker. Different genes within the sequence might be assigned to different lineages. (**c**) To establish a single sequence-level taxonomy, geNomad aggregates the lineages of all the markers using a weighted majority vote approach. This approach determines the support for each taxon at each taxonomic rank by summing the bitscores of all genes assigned to that taxon. The sequence is then assigned to the most specific taxon that is supported by at least 50% of the total bitscore of the sequence.

Sequences classified as viral with geNomad’s markers are then assigned to taxa defined by the International Committee on Taxonomy of Viruses (ICTV)^16^. This process is made possible by the fact that more than 85,000 of the markers are specific to a virus taxon (for more details, see ‘A dataset of marker protein profiles’ subsection). In brief, geNomad assigns a taxon to each gene annotated with a taxonomically informed marker. Subsequently, it aggregates the taxonomies of all the genes within each scaffold and generates a single consensus lineage for that sequence (Extended Data Fig. 3).

Upon completion of its execution, geNomad produces a list of sequences that have been classified as either plasmids or viruses. This list can be refined using additional user-adjustable filters, such as a minimum score, maximum FDR (if score calibration was performed), minimum number of plasmid or virus hallmark genes and maximum number universal single-copy genes. The generated output includes rich metadata that can be useful for downstream analysis (Fig. 1a, box A4) and the nucleotide and amino acid sequences of the identified plasmids and viruses.

### A dataset of marker protein profiles

**Extended Data Fig. 4:**
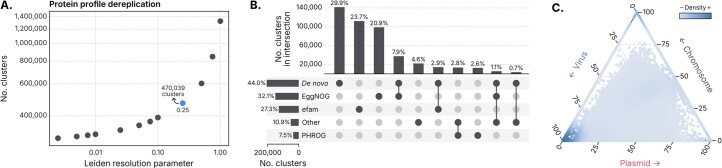
geNomad’s marker dataset was built by gathering dereplicated protein profiles from several sources and measuring their specificity to chromosomes, plasmids, and viruses. (**a**) Number of protein profile clusters obtained by varying the clustering granularity (Leiden’s resolution parameter). The value chosen for dereplication (0.25) is indicated in blue. (**b**) UpSet plot showing the overlap of different protein profile datasets in the dereplication process. The overlap between a given pair of datasets was measured as the number of protein profile clusters that contained profiles from both. (**c**) Ternary plot showing the specificity of protein profiles (circles) prior to dereplication (n = 470,039). Colors represent the marker density in a region of the plot.

geNomad uses a marker set of 227,897 protein profiles specific to chromosomes, plasmids or viruses to perform classification based on gene content and to provide functional information for processed sequences (Fig. 2a). To build this marker dataset, which covers sequences from uncultured microorganisms and viruses from diverse environments, we clustered approximately 232 million protein sequences from diverse sources (see ‘Database of genomic sequences for training and benchmarking’ section). The resulting clusters were independently aligned, generating 812,511 de novo protein profiles, which were further supplemented with 612,966 external profiles. To improve geNomad’s computational efficiency and ensure broad coverage of the gene space, we identified and removed redundant profiles, resulting in a collection of 470,039 non-redundant profiles (Extended Data Fig. 4a,b).

To select profiles that are informative for classification, we computed the specificity of each profile to each one of the targeted classes (chromosomes, plasmids and viruses) by mapping them to proteins encoded by reference genomes of both isolate and uncultivated species (Extended Data Fig. 4c) and counting the hits to each class. To mitigate the bias resulting from uneven taxonomic representation of plasmid and virus sequences in public databases, which favor elements infecting a limited range of microbes, we downweighted sequences belonging to overrepresented taxa by clustering them into reference clusters (RCs) that group similar genomes. We assigned weights to the references so that the sum of the weights in all RCs was constant, effectively downweighting sequences within large RCs^7^. After computing specificity, we discarded profiles that were poorly specific or that matched few proteins, resulting in a final set of 227,897 profiles. Most of the markers originated from the de novo protein clustering (38.8%), efam^17^ (34.9%) and EggNOG^18^ (16.0%) (Fig. 2b, top, and Supplementary Table 2). Virus-specific markers dominate the dataset (69.2%), followed by chromosome-specific markers (23.5%) and plasmid-specific markers (7.3%) (Fig. 2b, middle, lighter shades).

geNomad also provides detailed taxonomic and functional information for biological interpretation of results, enabling thorough analysis of identified MGEs. To allow this, markers were functionally annotated via alignment to the Pfam-A^19^, TIGRFAM^20^, KEGG Orthology^21^ and COG^22^ databases. In total, 98,127 (43.1%) markers were annotated, although the proportion of annotated markers varied among the different specificity classes, with chromosome-specific markers having the highest annotation rate (82.5%), followed by plasmid-specific markers (63.4%) and virus-specific markers (27.5%) (Fig. 2b, middle, darker shades, and Supplementary Table 2). Functional enrichment analysis of the annotated markers (Fig. 2c) revealed that chromosome markers were associated with translation, transport and metabolism functions; plasmid markers were enriched in quorum sensing and motility functions; and virus markers were related to virus replication and assembly functions. A total of 978 plasmid and 14,635 virus markers were manually selected as hallmark markers, as they were annotated with functions related to core processes, such as conjugation genes for plasmids and capsid proteins for viruses. To provide additional context for MGE research, markers were also annotated using databases for specific domains of interest (Supplementary Table 2), resulting in the identification of 484 markers for genes involved in conjugation and 382 markers for antimicrobial resistance, annotated through alignment with the CONJscan^23^ and NCBIfam-AMRFinder^24^ databases, respectively. Lastly, 741 markers for universal single-copy genes, which are rarely present in MGEs and can help reduce false positives, were identified through comparison with profiles from the BUSCO dataset^25^.

To allow taxonomic assignment of viruses using geNomad’s markers, virus taxa from the ICTV (Virus Metadata Resource version 19) were assigned to 85,315 markers. The taxonomically informed markers can be used to assign virus sequences to a substantial fraction of the viral taxa up to the family rank (Fig. 2b, bottom), as at least one marker was assigned to 83.3% of the realms (the only realm missing is *Ribozyviria*), 100% of the kingdoms and phyla, 94.9% of the classes, 87.7% of the orders and 61.8% of the families. Most of these markers were assigned to the *Caudoviricetes* class (93.1%), which dominates metagenomic data^9^, but other major taxa, such as *Riboviria* (2.8%), *Nucleocytoviricota* (2.2%) and *Monodnaviria* (0.7%), are also largely covered (Supplementary Table 2).

Our marker selection process was designed to maximize the range of covered uncultivated genomes found globally. To assess the environmental breadth of geNomad’s markers, we used them to scan a total of 2.3 billion proteins from 28,865 metagenomes and 7,258 metatranscriptomes of various ecosystems. The ecosystem distributions of the marker classes (chromosome-, plasmid- and virus-specific) were then evaluated (Supplementary Methods), revealing that chromosome-specific and plasmid-specific markers are generally not specific to any ecosystem (high average entropy of frequencies), whereas virus-specific markers tend to be restricted to specific ecosystems (low average entropy of frequencies) (Fig. 2d). This suggests that the gene repertoire of uncultivated viruses is highly variable and highlights the importance of incorporating environmental data to cover a large fraction of the virosphere.

### geNomad accurately identifies plasmids and viruses

**Extended Data Fig. 5:**
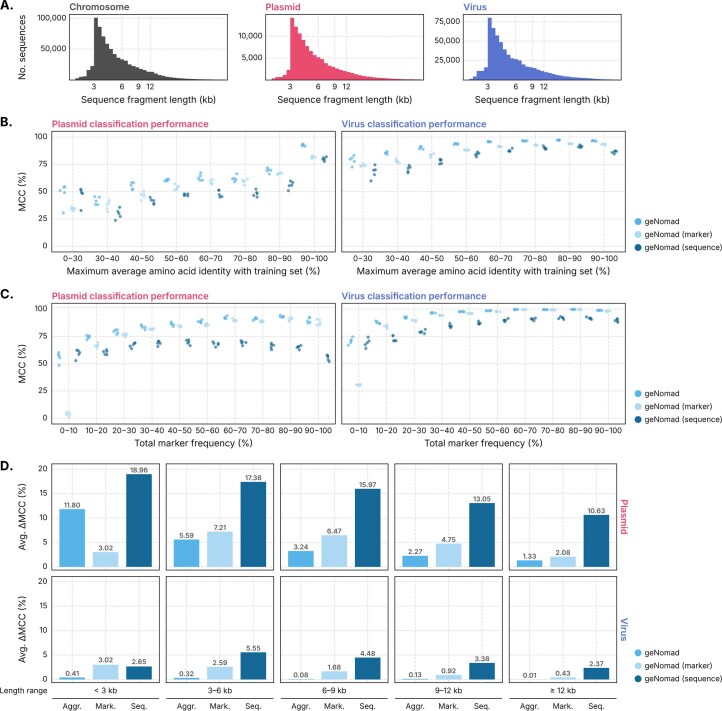
geNomad can detect plasmids and viruses with low identity to the training data even if they encode few or no markers. (**a**) Length distributions of the sequence fragments used to train geNomad and to evaluate classification performance of multiple tools. Sequence length (*x*-axis) is represented in log scale. (**b**) geNomad’s classification performance on plasmids (left) and viruses (right) with varying degrees of similarity to sequences in the train data (bins in the *x*-axis). Similarity to the train data was assessed by computing average amino acid identities to the sequences in the train data. (**c**) geNomad’s classification performance on plasmids (left) and viruses (right) with varying marker frequency (fraction of genes assigned to a geNomad marker). For each interval, performance was measured across five pairs of train/test sets (leave-one-group-out strategy). (**d**) Score calibration improves classification performance for both plasmids and viruses across all length ranges. Classification performance was measured using the Matthews correlation coefficient (MCC).

To evaluate the classification performance of geNomad and compare it to other virus and plasmid identification tools that use different approaches for sequence classification (Table 1), we used test datasets consisting of diverse sequence fragments with varying lengths (Extended Data Fig. 5a). To minimize overestimation of geNomad’s performance due to the presence of similar sequences in the train and test data, we randomly assigned RCs to five different data splits and performed cross-validation using the leave-one-group-out strategy (see Methods for details), which forced sequences from the same RC to remain together in either the train or test sets. Performance metrics for all tools were measured five times, using each RC as the test set at a time. Additional benchmark results are described in Supplementary Note 3.

**Table 1:** Classification methodology and average runtimes of plasmid and virus identification tools

Tool	Method	Plasmid	Virus/provirus	Runtime ± s.e.m. (s)
geNomad	Hybrid	✓	✓/✓	241.73 ± 0.18
geNomad (marker branch)	Marker-based	✓	✓/✓	119.20 ± 0.12
geNomad (sequence branch)	Alignment-free (IGLOO)	✓	✓/✗	118.58 ± 0.10
DeepMicroClass	Alignment-free (CNN)	✓	✓/✗	71.49 ± 0.13
DeepVirFinder	Alignment-free (CNN)	✗	✓/✗	710.00 ± 1.75
Phigaro	Marker-based	✗	✗/✓	1,585.61 ± 3.23
PlasClass	Alignment-free (k-mer freq.)	✓	✗/✗	20.50 ± 0.09
PlasX	Marker-based	✓	✗/✗	1,965.15 ± 1.02
PPR-Meta	Alignment-free (CNN)	✓	✓/✗	374.41 ± 1.21
Seeker	Alignment-free (LSTM)	✗	✓/✗	758.85 ± 2.93
VIBRANT	Marker-based	✗	✓/✓	662.35 ± 9.39
viralVerify	Marker-based	✓	✓/✗	1,641.64 ± 8.12
VirSorter2	Marker-based	✗	✓/✓	6,303.27 ± 4.25
VirSorter2 (all models)	Marker-based	✗	✓/✓	6,745.52 ± 10.14

Runtimes were measured across five executions using the hyperfine tool. A random selection of 10,000 metagenomic scaffolds (total of 18.3 megabases, IMG/M Taxon Object ID: 3300038405) was used as input for all tools. All speed measurements were performed in an Amazon EC2 instance (c5.12xlarge, SSD storage). Checkmarks indicate that the software is able to identify a given type of element (as indicated by the column name), while crosses indicate that the software can’t identify that type of element. CNN, convolutional neural network; freq., frequency; LSTM, long short-term memory neural network.

**Fig. 3:**
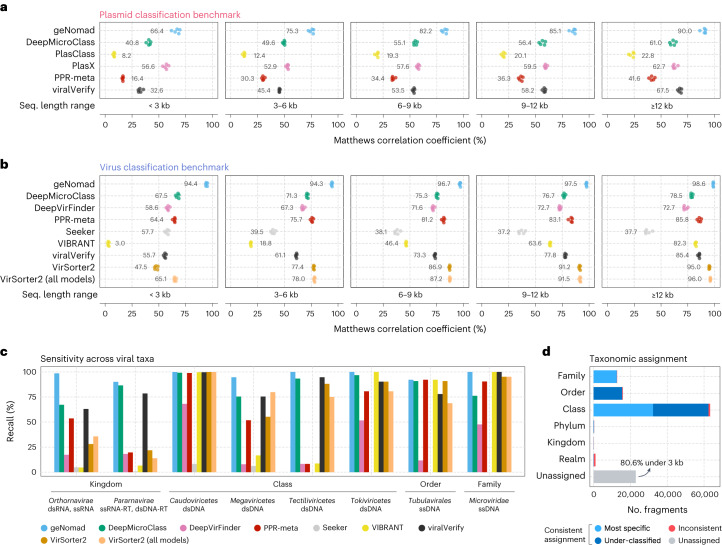
geNomad accurately identifies viruses and plasmids and allows taxonomic assignment of viral genomes. **a**,**b**, Classification performance of multiple plasmid (**a**) and virus (**b**) identification tools across sequence fragments of varying length. Performance was measured using the MCC. For each sequence range interval, tools were evaluated with five different test sets, each containing the sequences of one RC. Colored circles represent the performances measured in each test set. Mean values are shown next to the circles. **c**, Sensitivity of virus identification tools across major viral taxa at different ranks. The score cutoff of each tool was determined so that the FDR was approximately 5%. **d**, Virus taxonomic assignment performance. Bar lengths represent the number of sequence fragments assigned at a given taxonomic rank. Light blue represents sequences that were correctly assigned to their most specific rank (up to the family level); dark blue represents fragments that were assigned to the correct lineage but to a rank that is above its most specific rank; red represents sequences that were assigned to the wrong lineage; and the gray bar represents sequences that were assigned to any taxon.

By evaluating the classification, measured using the Matthews correlation coefficient (MCC), as a function of the similarity to the train data, we found that geNomad performs well on unseen genomes, even though performance dropped for sequences that were more divergent from the train data (Extended Data Fig. 5b). Assessment of geNomad’s performance on sequences with varying marker coverage (that is, fraction of proteins assigned to markers) revealed that even those that were targeted by no or few markers were still detected due to the sequence branch of the algorithm (Extended Data Fig. 5c). When compared to other tools, geNomad presented superior overall classification performance across all sequence length ranges in both plasmid and virus classification tasks (Fig. 3a,b and Supplementary Tables 3 and 4). Such difference was particularly apparent for short sequences (<6 kilobases (kb)), where other tools showed reduced performance due to limited genetic information, whereas geNomad leveraged its extensive marker dataset and alignment-free classification model, ensuring high sensitivity and precision. This highlights the usefulness of geNomad in metagenomic and metatranscriptomic assemblies, where most scaffolds are short.

geNomad’s calibration mechanism enhances the classification process by incorporating sample composition data and assigning estimated probabilities to each sequence, which reflect the likelihood of the sequence belonging to each class. Our analysis showed that the plasmid classification performance increased with the use of calibrated scores, particularly for shorter sequences (average ΔMCC: +11.8% for sequences <3 kb; +5.6% for 3–6 kb; and +3.2% for 6–9 kb) (Extended Data Fig. 5d). We also found that short virus sequences benefited from calibration, although the improvement was not as pronounced. These results showcase the effectiveness of the introduced calibration mechanism for improving classification quality.

Plasmid classification is a challenging task due to the variable genetic makeup of these elements, their similarity to other mobile elements that can integrate into host chromosomes and the lack of a standard for reporting plasmids in sequencing data. As a result, most evaluated tools (DeepMicroClass^26^, PPR-Meta^27^, PlasClass^28^ and viralVerify^29^) had low average classification precision (11.0–40.1%; Supplementary Table 3), even when classifying long sequences (Supplementary Table 4), as they often produced a high number of false positives that can impact downstream analysis. In contrast, PlasX^7^ had high precision (81.6%) but low sensitivity (40.5%), which impairs the detection of plasmids in sequencing data. geNomad had the best overall performance by a substantial margin (Fig. 3a; MCC and F1-score in Supplementary Tables 3 and 4), with the highest sensitivity (89.8%) and the second highest precision (70.8%), after PlasX. It is worth noting that geNomad’s marker branch, which can be run independently, achieved a considerably higher precision than PlasX (91.2%). Evaluation of classification performance across diverse taxa revealed that geNomad outperformed other tools in all assessed groups (Supplementary Table 5 and Supplementary Note 3). Furthermore, geNomad exhibited a lower rate of misclassifying viruses as plasmids (1.7%) compared to all tools except PlasX (1.5–64.4%; Supplementary Table 6 and Supplementary Note 3).

In virus classification, geNomad attained the best overall performance when considering all length strata (MCC: 95.3%, F1-score: 97.3%), followed by VirSorter2 (ref. ^30^) executed with all models (MCC: 81.3%, F1-score: 88.9%), VirSorter2 executed with default parameters (MCC: 79.7%, F1-score: 87.1%) and PPR-Meta (MCC: 77.4%, F1-score: 86.6%) (Fig. 3b and Supplementary Table 3). VIBRANT^31^, geNomad, VirSorter2 (default parameters) and DeepMicroClass achieved the highest classification precision (97.5%, 97.3%, 94.7% and 92.6%, respectively), and Seeker^32^, DeepVirFinder^33^ and PPR-Meta obtained the lowest scores (61.8%, 80.5% and 88.5%, respectively).

In a benchmark study using representative genomes from the ICTV, we found that geNomad outperformed other tools in all major taxa that we evaluated (Fig. 3c and Supplementary Table 7). Notably, geNomad was the only tool that achieved high sensitivity for viruses that encode an RNA-dependent RNA polymerase (RdRP; *Orthornavirae*, 98.64%) and giant viruses (*Megaviricetes*, 94.74%) at a fixed FDR of 5%. When evaluating sensitivity across different host clades, we found that geNomad was the only tool that identified more than 90% of the viruses infecting bacteria, archaea and multiple eukaryotic groups, whereas other tools struggled to identify viruses that infect at least two eukaryotic groups (Supplementary Table 8). In an additional benchmark where we measured classification sensitivity on a catalog of metagenomic *Inovirus*^34^, which are known to be challenging to detect automatically, geNomad (sensitivity: 84.8%) also outperformed other evaluated tools (average sensitivity: 32.5%) (Supplementary Table 9).

We assessed the performance of geNomad’s taxonomic assignment (Fig. 3d and Supplementary Table 10) by assigning 116,250 artificially fragmented genomes of ICTV exemplar species to viral lineages using a marker dataset with modified taxonomic metadata to simulate novelty (see Methods for details). Of the processed fragments, the majority (80.3%) was successfully assigned to a viral lineage, with most being classified at the class (54.4%), order (13.6%) or family (10.1%) levels. Among those, 48.2% were correctly assigned to the most specific rank (up to the family level); 49.5% were under-classified (assigned to the correct lineage but not to the most specific rank); and only 2.3% were assigned to the wrong lineage. These results indicate that geNomad is reliable at assigning sequences to higher taxa. The unassigned fragments, which lacked hits to markers with taxonomic information, were mostly shorter than 3 kb (80.6%).

### Sensitive and precise identification of proviruses

**Fig. 4:**
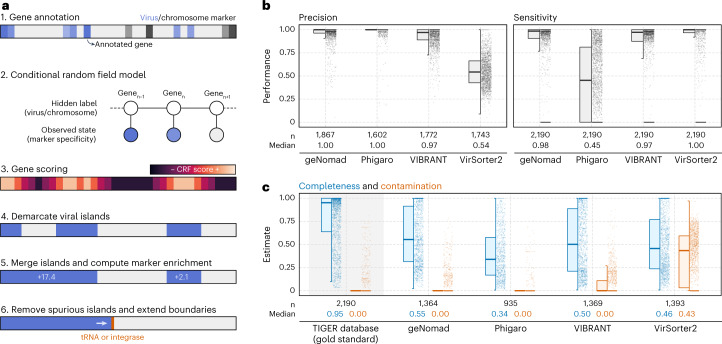
geNomad uses marker information to demarcate provirus boundaries. **a**, Provirus identification starts by annotating the genes within a sequence with geNomad markers, which store information of how specific they are to hosts or viruses. These specificity values are then fed to a CRF model, which will score each gene using information from the markers in its surroundings. A score cutoff is used to demarcate viral islands, and islands that are close together are merged. Islands with few viral markers are discarded, and the boundaries of the remaining islands are extended up until nearby tRNAs or integrases. **b**, Distributions of the precision and sensitivity of multiple provirus identification tools, measured at the gene level for each provirus. Proviruses from the TIGER database were used as the ground truth for this benchmark. **c**, Completeness and contamination estimates of demarcated proviral regions that did not overlap with proviruses in the TIGER database. Estimates for TIGER proviruses are shown with a gray background as a reference. Box plots show the median (middle line), interquartile range (box boundaries) and 1.5 times the interquartile range (whiskers).

Temperate phages can integrate into host genomes and form proviruses, which can greatly affect host metabolism and ecology^35–37^. To identify integrated viruses within host genomes, geNomad employs a conditional random field (CRF) model that identifies genomic regions that exhibit a high enrichment of viral markers and are flanked by chromosome markers (Fig. 4a). The CRF model leverages the extensive gene coverage provided by the marker database and scores each gene, factoring in the specificity levels of assigned markers for that gene and its neighboring genes. To eliminate spurious viral islands (regions of consecutive genes labeled as viral), geNomad merges closely located islands and subsequently removes those with a low marker enrichment—that is, regions containing only a few virus markers. Finally, because tRNAs and integrases are commonly found next to the edges of integrated elements due to the dynamics of site-specific recombination^38^, geNomad extends provirus boundaries up until neighboring tRNAs and/or integrases, improving the detection sensitivity of genes close to provirus edges.

**Extended Data Fig. 6:**
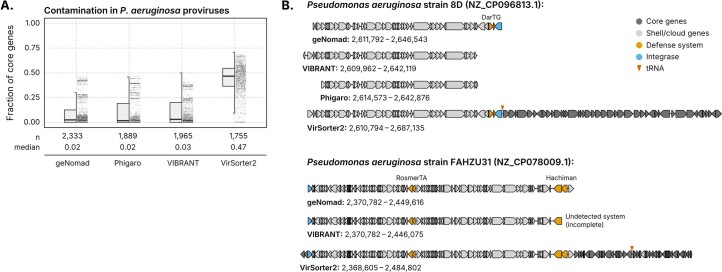
geNomad outperforms other tools in identifying proviruses in the *Pseudomonas aeruginosa* pangenome. (**a**) Distribution of the contamination estimates of multiple provirus-identification tools, measured at the gene-level for each provirus. Contamination was measured as the number of core genes, as determined by PPanGGOLiN, in the provirus. The number of detected provirus and the median contamination of each tool are displayed below the graph. Box plots show the median (middle line), interquartile range (box boundaries), and 1.5 times the interquartile range (whiskers). (**b**) Defense system-encoding proviral regions demarcated with multiple tools in *P. aeruginosa* genomes. Shell and cloud genes are shown in light grey and core genes (putative contamination) are shown in dark gray. Genes that are part of defense systems are in orange. Integrase genes are in blue. tRNA loci are indicated by red arrows. GenBank accessions are shown within parenthesis. Phigaro did not detect any provirus within the 2,370,782–2,449,616 bp region in the NZ_CP078009.1 sequence.

We evaluated geNomad’s provirus demarcation performance and compared it with other popular tools (Phigaro^39^, VIBRANT and VirSorter2) using the TIGER dataset^38^, which contains precisely mapped integration sites across 2,168 prokaryotic genomes, as the ground truth (Fig. 4b and Supplementary Table 11). For each predicted proviral region by the benchmarked tools, we measured precision as the fraction of genes within TIGER proviruses and sensitivity as the proportion of genes contained within regions predicted by each tool. The results of this benchmark demonstrated that geNomad identified more proviruses than other tools and exhibited high precision and sensitivity. Not all the predicted proviral regions overlapped with TIGER coordinates, because this dataset does not include inactive phages nor proviruses that do not integrate at tRNAs. To measure the quality of such predictions, we used CheckV^40^ (version 1.0.1) to estimate the quality of these regions and found that geNomad outperformed other tools, as the proviruses it demarcated tended to be more complete with lower contamination levels (that is, few host genes) (Fig. 4c and Supplementary Table 11). The completeness of most of these proviral regions was comparatively lower than those in TIGER, indicating that they likely represent inactive proviruses that underwent gene loss. In an additional benchmark, we found that geNomad outperforms other tools in the identification of proviruses in a *Pseudomonas aeruginosa* pangenome^41^ (Supplementary Note 4, Extended Data Fig. 6 and Supplementary Table 11).

### geNomad is fast and allows analysis of large datasets

To make geNomad accessible to a wide audience, we designed it to be user-friendly and efficient, allowing it to run quickly on a broad range of hardware. geNomad can be installed locally though diverse methods (pip, Conda and Docker), facilitating its installation in a variety of scenarios. The command line interface offers comprehensive explanations and detailed execution logging. For non-technical users, geNomad is available as a web application through the NMDC EDGE platform (https://nmdc-edge.org/virus_plasmid/workflow), allowing easy data upload and result visualization in the web browser. Additionally, the integration with NMDC EDGE enables geNomad to be easily incorporated into larger workflows that include other tasks, such as assembly and binning.

In a benchmark measuring the time it took to classify 10,000 metagenomic scaffolds, geNomad was faster than all but two of the evaluated tools (Table 1), taking substantially less time than VirSorter2 (26.1× improvement), PlasX (8.1×), viralVerify (6.8×) and VIBRANT (2.7×). The only tools that were faster were DeepMicroClass and PlasClass, which are alignment-free tools that exhibited lower classification performance than geNomad in our benchmarks (Fig. 3a). It is worth noting that geNomad’s marker and sequence branches can be run independently, reducing runtime by half while still maintaining good classification performance (Supplementary Table 3), in cases where time is a concern. These results demonstrate that, due to its speed, geNomad can be used in varied hardware and can be scaled to process large datasets. In fact, geNomad was recently used to process approximately 260 million scaffolds (2.7 trillion base pairs) from IMG/M to gather the data used to build the IMG/VR version 4 (ref. ^9^) and IMG/PR databases, which represent the largest available databases of virus and plasmid sequences, respectively.

### geNomad allows the discovery of RNA and giant viruses

Recent studies have unveiled a previously undiscovered diversity of RNA viruses (*Orthornavirae* kingdom) and giant viruses (*Nucleocytoviricota* phylum) through the analysis of sequencing data from metatranscriptomes and metagenomes^14,42–46^. As existing virus discovery tools exhibit limited efficacy in detecting a substantial fraction of the RNA and giant virus genomes (*Orthornavirae* and *Megaviricetes* in Fig. 3c), these large-scale surveys have resorted to custom techniques, such as identifying the RdRP hallmark gene for RNA viruses and employing metagenomic binning for giant viruses. However, these tailored approaches are often difficult to reproduce, as they were developed for internal use. To address this issue and increase the sensitivity of detecting both RNA and giant viruses in sequencing data, we leveraged recent knowledge about these viruses to train geNomad, which improved the identification of these lineages (Fig. 3c, Supplementary Note 5 and Supplementary Note 6).

**Fig. 5:**
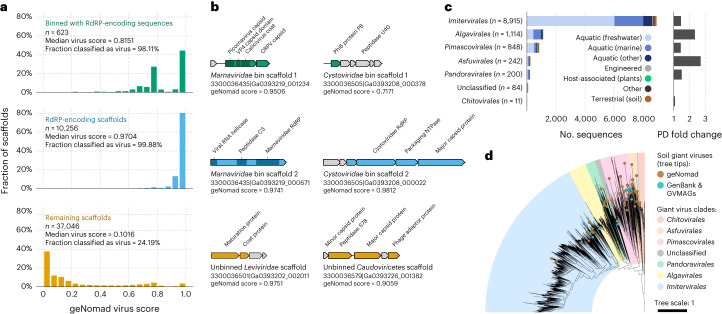
geNomad allows the discovery of RNA viruses and giant viruses in environmental sequencing data. **a**, Histograms showing the geNomad score distribution of three groups of scaffolds of the Sand Creek Marshes metatranscriptomes: scaffolds that binned with RdRP-encoding sequences (top row, in green); scaffolds that contain the RdRP gene (middle row, in blue); and the remaining scaffolds (bottom row, in orange). The median geNomad score and the fraction of scaffolds classified as viral are indicated for each group. **b**, Genome maps of selected sequences that were classified as viral by geNomad. Two pairs of co-occurring *Orthornavirae* scaffolds are represented (*Marnaviridae* and *Cystoviridae* bins). Genes targeted by geNomad markers are colored, and genes that do not match any marker are shown in gray. Rows and colors match those of **a**. **c**, Number of scaffolds assigned to *Nucleocytoviricota* orders across multiple ecosystems (left bar plot). Sequences were identified by geNomad in a large-scale survey of metagenomes of diverse ecosystems. Only scaffolds that are at least 50 kb long or more were evaluated. Bar colors represent the ecosystem types where the sequences were identified. The phylogenetic diversity (PD) fold change is shown on the right bar plot. PD fold change values correspond to the ratio between the total PD of trees reconstructed with and without geNomad-identified giant viruses. **d**, Maximum likelihood phylogenetic tree of soil giant viruses identified with geNomad (brown tree tips). Reference sequences from GenBank and from a previous metagenomic survey (GVMAGs) were included, and the ones that were sequenced from soil samples are indicated with turquoise tree tips. Tree tips that are not colored represent representative genomes sequenced from samples obtained from other ecosystems. The ranges corresponding to different *Nucleocytoviricota* orders are represented using distinct colors.

In metatranscriptomes from microbial communities of the Sand Creek Marshes^47^, geNomad classified 99.9% of the sequences containing the RdRP gene as viral (Fig. 5a). Furthermore, we found that 98.1% of the scaffolds that binned^48^ with RdRP-encoding sequences based on their co-occurrence across multiple samples were also identified as viral by geNomad. This indicates that geNomad can identify RNA virus genome sequences even when they lack the RdRP gene (Fig. 5a). In contrast, other tools classified an average of only 43.7% of these sequences as viral (Supplementary Table 12). Inspection of pairs of co-occurring scaffolds revealed that they fell into two categories: (1) linear genomes that were assembled into two scaffolds, one of which lacked the RdRP gene (*Marnaviridae* bin in Fig. 5b); and (2) segmented genomes, containing multiple DNA molecules (*Cystoviridae* bin in Fig. 5b). Among sequences not encoding RdRP and not binned with RdRP-encoding scaffolds, yet classified as viruses by geNomad, we found fragments of RNA virus genomes missing the RdRP gene (*Leviviridae* scaffold in Fig. 5b) and transcripts of DNA viruses (*Caudoviricetes* scaffold in Fig. 5b).

To assess geNomad’s capability to uncover new clades of giant viruses, we applied it to 28,865 metagenome assemblies from the IMG/M^49^ database. Scaffolds classified as virus by geNomad that were at least 50 kb in length were further analyzed using the GVClass pipeline, which placed *Nucleocytoviricota* scaffolds in a phylogenetic context by identifying a set of conserved protein families and reconstructing gene trees together with reference genomes. A total of 11,414 scaffolds identified by geNomad were phylogenetically placed in the *Nucleocytoviricota* tree (Fig. 5c and Supplementary Table 13). Other tools classified, on average, 77.4% of these scaffolds as viral (Supplementary Table 14). Within metagenomes from soils, an understudied niche for giant viruses^50^, we identified 235 additional *Nucleocytoviricota* scaffolds, up from 16 metagenomic bins reported in the previous survey. Phylogenetic reconstruction of these soil giant viruses revealed that they include several novel clades of *Imitervirales*, *Pimascovirales* and *Asfuvirales* that do not have representatives in GenBank or Schulz et al.^14^ (Fig. 5d), suggesting that the underlying diversity of *Nucleocytoviricota* in soil is greatly underestimated.

More information on the RNA and giant virus surveys can be found in Supplementary Notes 5 and 6. The methodology is detailed in Supplementary Methods.

## Conclusion

Identifying plasmids and viruses in sequencing data is a crucial process, as it sheds light on the diversity of these mobile elements, on their impact on the evolution and on ecological interactions of cellular organisms, and it facilitates high-throughput monitoring of clinically relevant strains. Here we present geNomad, a novel computational framework that enables the identification and annotation of plasmids and viruses in sequencing data. This is supported by a database of marker protein profiles that are richly annotated in terms of functional and taxonomic information and that serves as a valuable community resource that can be leveraged independently of geNomad (see the ‘Code availability’ and ‘Data availability’ sections for download information). As a result, this framework has broad application for sequence classification and annotation, allowing, for example, end-to-end identification of conjugative plasmids that carry AMR genes. geNomad incorporates innovative concepts, such as a hybrid classification process that combines alignment-free and gene-based approaches in a principled manner, and a score calibration algorithm that enhances the quality and interpretability of results. Given its improved classification performance and computational efficiency compared to other tools, as well as its ability to taxonomically classify viruses and functionally annotate genes, we anticipate that geNomad will be a valuable resource for the plasmid and virus research communities. We also foresee that it will drive further exploration of the virosphere and foster new initiatives to uncover the diversity and ecology of plasmids in natural environments, a topic that has often been overlooked.

## Methods

### Database of genomic sequences for training and benchmarking

Prokaryotic genomes (2,886 bacterial and 336 archaeal) were retrieved from GTDB^51^ (release 202). To mitigate taxonomic bias, we only used the genome with the highest quality score (completeness − 5 × contamination − 0.05 × no. scaffolds) per GTDB family. Provirus and provirus-like regions were identified and removed from the scaffolds using VirSorter2 (version 2.2.2), Phigaro (version 2.3.0) and VIBRANT (version 1.2.1). Plasmids were removed by identifying sequences containing the word ‘plasmid’ in their header or sharing at least half of their genes with any plasmid in the PLSDB database^52^ (release 2020_11_19). Eukaryotic genomes were obtained from the TOPAZ dataset^53^, which includes 988 metagenome-assembled genomes of small eukaryotes. To reduce taxonomic imbalance, we clustered TOPAZ genomes based on their amino acid identity (AAI) into 385 clusters using the Leiden algorithm^54^ (as implemented in the igraph Python package, resolution parameter = 0.5) and picked the genome with the least contamination, as estimated by the study’s authors, as the representative.

Plasmid sequences were obtained from the PLSDB database (release 2020_11_19), RefSeq (archaeal plasmids, retrieved on 23 July 2021) and a dataset of complete plasmids identified in metagenomic data (IMG/M Taxon Object ID: 3300053491). To identify chromosome sequences that were mislabeled as plasmids, we performed gene prediction with Prodigal (version 2.6.3, parameters: ‘-m -p meta’) and used hmmsearch^55^ (HMMER version 3.3.2, parameter: ‘--cut_ga’) to match the proteins to sets of single-copy genes (ar122 and bac120, from GTDB). Scaffolds encoding two or more single-copy genes were discarded.

To further remove viral scaffolds from the prokaryotic and eukaryotic chromosome datasets, as well as phage plasmids from the plasmid data, we performed an additional filter using HMMs of viral hallmarks from VirSorter2 and viral and host markers from CheckV (database version 1.0). In brief, we used hmmsearch (parameter: ‘-E 1e-5’) to match Prodigal-predicted proteins from all chromosome and plasmid scaffolds to these HMMs and discarded the sequences that encoded any viral hallmark or that had no. viral markers ≥0.5 × no. host markers.

The virus sequence dataset was assembled using data from GenBank (retrieved on 6 July 2021), IMG/VR version 3 (ref. ^56^) *Nucleocytoviricota* from Schulz et al.^14^, *Leviviridae* from Callanan et al.^57^, Asgard archaea viruses from Medvedeva et al.^58^, archaeal tailed viruses from Liu et al.^59^ and *Orthornavirae* from Neri et al.^44^. To remove short genome fragments and contaminants from the IMG/VR sequences, we retained only sequences that contained direct terminal repeats or that fulfilled the requirements to be considered high quality according to the MIUViG standard^60^. Because the *Nucleocytoviricota* genomes from Schulz et al. consist of metagenomic bins that might contain contamination, we opted to keep only the contigs that encode the major capsid protein (MCP), identified using hmmsearch (parameter: ‘-E 1e-5’) to match their proteins to the set of MCP HMMs provided in the original study.

To reduce sequence redundancy, plasmid and virus scaffolds were de-replicated using pairwise average nucleotide identities (ANIs), computed as described in Nayfach et al.^40^ (code available at https://bitbucket.org/berkeleylab/checkv/src/master/scripts/anicalc.py). Specifically, we used MegaBLAST^61^ (version 2.11.0+) to perform all-versus-all nucleotide alignments and computed the pairwise ANI as the length-weighted average identity of all the matches between a pair of sequences. Next, scaffolds with ANI ≥ 97% over at least 95% of the length of the shorter sequence were clustered using a greedy algorithm^62^, and the longest sequence within each cluster was selected as the representative. Scaffolds shorter than 2,000 bp were discarded. The final selection contained 300,990 sequences from prokaryotic chromosomes, 42,595 sequences from eukaryotic chromosomes, 41,424 plasmid sequences and 240,411 virus sequences.

To account for the taxonomic representation imbalance of public databases, plasmid and virus sequences were structured into RCs containing related sequences. RCs would serve two purposes: (1) to minimize representation bias in model training, by downweighting the sequences within large RCs so that the total weight within each RC was the same; and (2) to allow informed cross-validation splits^7^, where the sequences of a given RC will remain together in either the train or test sets, allowing us to measure geNomad’s performance on novel genomes. To obtain the RCs, we computed the AAIs between all pairs of plasmids and viruses and built a graph using these values as edge weights (code available at https://github.com/apcamargo/bioinformatics-snakemake-pipelines/tree/main/contig-aai-pipeline). Next, we employed the Leiden algorithm to cluster the sequences, tuning the resolution parameter to make the average within-cluster AAI close to 95%. In total, we obtained 32,134 plasmid RCs and 215,618 virus RCs. Because prokaryotic and eukaryotic scaffolds are organized in genomes, we treated all the sequences within a given genome as members of the same RC. The RCs were randomly assigned to five distinct data splits that would be used for benchmarking.

Given that metagenomic assemblies mostly comprise short sequence segments, we created a dataset of artificially fragmented sequences that would be used for model training and evaluation. We first built an empirical length distribution from all public IMG/M metagenomes (as of 11 September 2021) and truncated the distribution to a minimum of 3,000 bp. Next, we split the sequences of our final selection into fragments whose lengths were randomly drawn from the distribution. Sequences shorter than 3,000 bp were left untouched.

Across all analyses, AAI was computed using Prodigal (version 2.6.3, parameters: ‘-m -p meta’) to perform protein prediction and DIAMOND^63^ (version 2.0.15, parameter: ‘--sensitive’) to carry out all-versus-all protein searches. Pairwise AAI values were computed as the length-weighted average identity of the reciprocal best hits of pairs of scaffolds that share at least 75% of the proteins of the shortest sequence. Only matches with E-value ≤ 0.001 and query and target alignment coverage ≥50% were allowed.

### Marker protein profile database

To build a comprehensive dataset of protein profiles that could be used to identify diverse plasmids and viruses, as well as to identify provirus boundaries, we gathered protein alignments from external sources and built de novo clusters from a diverse collection of protein sequences. Alignments were retrieved from the following external sources: Pfam-A seed alignments (release 34.0), TIGRFAM (release 15.0), ECOD^64^ (release 20210713), EggNOG Bacteria/Archaea/Virus (version 5), VOGdb (release 206, retrieved from https://vogdb.org/), PHROG^65^, efam and efam-XC, CONJscan, double jelly-roll MCPs from Yutin et al.^66^, *Lavidaviridae* MCPs and core proteins from Paez-Espino et al.^67^, *Inoviridae* protein families from Roux et al.^34^, *Leviviridae* core proteins from Callanan et al.^57^ and RdRPs from the RVMT dataset^44^.

De novo protein clusters were built from 232,031,767 protein sequences retrieved from IMG/VR version 3, GTDB (release 202) species representatives, GenBank viruses (retrieved on 6 July 2021), PLSDB (release 2020_11_19) and complete metagenomic plasmids (IMG/M Taxon Object ID: 3300053491). We first de-replicated these proteins at 95% identity using MMseqs2 linclust^68^ (version 13-4511, parameters: ‘--kmer-per-seq 80 -c 1.0 --cluster-mode 2 --cov-mode 1 --min-seq-id 0.95’). Next, we clustered the de-replicated protein sequences with MMseqs2 cluster, requiring a minimum 80% bidirectional alignment coverage (parameters: ‘-s 5.5 -e 1e-5 -c 0.8 --cov-mode 0 --cluster-mode 0 --max-seqs 5000 --min-seq-id 0.5 --cluster-reassign 1’). Finally, we performed multiple sequence alignment of the 786,782 clusters containing at least 20 proteins using Kalign^69^ (version 3.3.1). To improve the coverage of target viral groups, we performed independent clustering of the proteins obtained from the *Nucleocytoviricota* from Schulz et al.^14^, Asgard archaea viruses from Medvedeva et al.^58^, archaeal tailed viruses from Liu et al.^59^ and unannotated domains of polyproteins from the RVMT dataset. For these datasets, we allowed clusters to contain as few as four proteins.

To identify the protein profiles that are informative for sequence classification (hereafter, markers), we measured the specificity of the 1,425,477 profiles by computing the weighted number of matches of each profile to each class (chromosome, plasmid and virus). We first assigned weights to each sequence in such a way that the total weight of each RC within each class would be the same and that the total weight of the three classes would also be identical. Next, we converted the protein profiles into HMMs and used hmmsearch (parameter: ‘-E 1e-5’) to match them to Prodigal-predicted proteins from the sequence dataset. Finally, we counted the number of matches of each profile to each class, taking into account the RC weights and scaled the counts within each class so that the median profile count would be the same for the three classes. Scaled counts were used to compute each profile’s Pielou’s specificity—a single summary of the profile’s specificity—and specificity measures (SPMs)—which measure how specific the profiles are to each class—using tspex^70^ (version 0.6.2).

To reduce the redundancy of the protein profile set, we first used the HMMs to generate artificial protein sequences with the hmmemit command (parameter: ‘-N 10’) and then used hmmsearch (parameter: ‘-E 1e-5’) to align the HMMs to all artificial protein sequences. Next, to measure the empirical redundancy of all possible pairs of protein profiles, we employed SetSimilaritySearch (version 0.1.7, available at https://github.com/ekzhu/SetSimilaritySearch) to compute the cosine similarity of all pairs of profiles, based on the identity of their hits. Finally, we identified groups of profiles targeting similar protein sets by clustering them with the Leiden algorithm (resolution parameter = 0.25). The most specific profile in each cluster, determined by Pielou’s specificity, was selected as its representative.

To select the markers that would be used for classification, we identified protein profiles that had either Pielou’s specificity ≥0.4 or the maximum SPM (among the three classes) ≥0.75. For chromosome markers, we required highly prevalent profiles, above the median count distribution, to avoid selecting markers that target genomic island, which are enriched in mobile elements. For plasmid and virus markers, we required profiles to be above the first quartile of the distribution. To address misclassification of eukaryotic sequences as viral, we negatively selected virus-specific profiles that frequently matched eukaryotic proteins. Our approach involved retrieving eukaryotic proteins belonging to ortholog groups from OrthoDB^71^ (version 10.1) and removed the ones that corresponded to typical viral genes, resulting in a total of 16,928,157 eukaryotic proteins. We also obtained the sequences of 159,003 proteins that were shown to have been horizontally transferred from viral to eukaryotic genomes^72^. By employing hmmsearch, we matched HMMs of virus-specific markers to these eukaryotic proteins and removed profiles with at least 200 matches to OrthoDB proteins or at least 10 hits to horizontally transferred proteins. Ultimately, 227,897 profiles were selected to be used in geNomad for distinguishing among chromosome, plasmid and virus sequences. For benchmark purposes, we repeated this process five additional times, using only the train sequences of each data split to perform the selection.

To assign functional annotations to the geNomad protein profiles, we used HHblits^73^ (version 3.3.0) to align them with HMMs from Pfam-A (release 35.0), TIGRFAM (release 15.0), KEGG Orthology (release 98.0), COG (release 2020), CONJscan, NCBIfam-AMRFinder (release 2022-10-11.2) and *Bacteria* and *Archaea* near-universal single-copy orthologs from BUSCO (version 5). We accepted hits with probability ≥90%, E-value ≤ 0.001 and target coverage ≥60%. For Pfam, multiple non-overlapping hits were allowed, whereas only the best hit was retained for other databases. Names and Gene Ontology (GO) terms were assigned to geNomad markers by transferring them from the accepted Pfam, TIGRFAM and KEGG Orthology hits. GO enrichment for each class was appraised using the Kolmogorov–Smirnov test (as implemented in the hypeR^74^ package, version 1.13.0; FDR < 0.01) on lists of markers sorted by the SPM of each class. REVIGO^75^ was used to generate visualizations of the enriched GO terms.

To assign ICTV taxa to geNomad markers, we first built a protein database from viral sequences retrieved from NCBI NR (on 19 May 2022) and decorated the proteins with a custom taxdump generated from ICTV’s VMR 19 using TaxonKit^76^ (version 0.11.1). We then used MMseqs2 to align geNomad’s markers to the viral protein database (parameters: ‘-s 8.2 -e 1e-3’) and employed taxopy (version 0.9.2, available at https://github.com/apcamargo/taxopy) to assign a taxon to each marker by aggregating the taxonomic lineages of all the hits of each marker using the ‘find_majority_vote’ function. Because viruses of the *Nucleocytoviricota* phylum encode homologs of bacteriophage proteins^77^, we raised the minimum fraction parameter to 0.85 to assign taxonomy to markers that were initially assigned to *Nucleocytoviricota* but matched at least one *Caudoviricetes* protein. For benchmarking purposes, we simulated taxonomic novelty by masking proteins that had ≥60% identity to proteins of exemplar species.

### Classification models

To train the gene-based classifier, we first predicted the proteins encoded by the sequence fragments using prodigal-gv (version 2.7.0, parameter: ‘-p meta’, available at https://github.com/apcamargo/prodigal-gv). Next, we assigned geNomad markers to the predicted proteins using MMseqs2’s protein profile (parameters: ‘-s 6.4 -e 1e-3 -c 0.2 --cov-mode 1’). For each sequence, we computed a total of 25 features derived from the gene structure and marker annotation (full list and description in Supplementary Note 1) and used them to train a decision forest classification model with the XGBoost^78^ library (version 1.5.1, parameters: ‘eta=0.2, max_depth=10, n_estimators=135’). Feature selection was performed using the Boruta algorithm and SHAP importance values, as implemented in the shap-hypetune package (version 0.2.4, ‘BoostBoruta’ function). Hyperparameter tuning (learning rate, tree depth and number of trees) was performed using grid search (‘BoostSearch’ function in shap-hypetune).

The sequence-based classifier was trained using a two-step supervised contrastive learning approach^79^ (Extended Data Fig. 1). In the first step, we trained an IGLOO encoder to learn to produce vector representations of nucleotide sequences in such a way that sequences of the same class will tend to be clustered together and separate from sequences of different classes. In the second step, we trained a dense neural network classifier on top of the IGLOO representations using a focal loss^80^, which forces the model to focus on hard-to-classify sequences. Training was conducted using the Adam optimizer with gradient centralization^81^. Hyperparameter tuning was performed with KerasTuner (version 1.1.0) using the HyperBand algorithm^82^. For further details regarding the architecture and training process of the alignment-free classification model, see Supplementary Methods.

The outputs of the gene-based and sequence-based classifier are aggregated by a feedforward neural network, which uses an attention mechanism to weight the contribution of each model toward the final scores. In brief, we trained a model that encodes in an attention matrix *A* the reliability of the gene-based classifier, estimated from the relative marker frequency within each sequence. To aggregate the results of the two classifiers, the scores generated by them are scaled according to their expected reliability encoded in *A* and then averaged and fed to a dense layer with softmax activation.

For benchmark purposes, we trained the gene-based classifier, the sequence-based classifier and the aggregator model five additional times, using the train data and the selected markers of each data split. The models used for the remaining analysis were trained with the entire dataset.

### Score calibration model

To train the model underlying geNomad’s score calibration, 1,000,000 artificial communities with varying proportions of chromosome, plasmid and virus sequences were generated by random sampling of the train dataset. For each community, scores were calibrated using an isotonic regression, and the empirical composition was obtained by using geNomad to predict the most likely class of each sequence. Because isotonic regressions are dataset specific, a regression feedforward neural network was trained to predict calibrated scores from the empirical composition and uncalibrated scores of a given community. The model was trained with the Adam optimizer and mean squared error loss.

### Provirus identification

To identify regions that correspond to putative proviruses within host chromosomes, geNomad employs a CRF model that was trained on a dataset of mock proviruses built from prokaryotic chromosome sequences and phage genomes. The CRF takes as input the chromosome and virus SPM values of the genes annotated with geNomad markers and computes the conditional probability of a sequence of states (chromosome or provirus). Genes are then assigned to their most likely states, forming provirus islands—that represent regions that are enriched in virus markers. To prevent having proviruses split into multiple islands due to incomplete marker coverage, provirus islands that are separated by short gene arrays (fewer than six genes or two chromosome markers) are merged. Next, provirus boundaries are refined by extending them to the closest tRNA (identified with ARAGORN^83^, version 1.2.41) within 5 kb and integrase (identified using MMseqs2 profile search) within 10 kb, as long as there are no chromosome markers between the original edge and the new putative coordinate. The 16 tyrosine integrase profiles used for integrase identification were manually selected from the CDD database^84^. Finally, islands with few viral markers, which usually are not bona fide proviruses, are filtered out by removing the regions where the sum of the virus SPM of the markers is below a certain threshold.

### Performance benchmarks

The following tools were included in our benchmarks: geNomad (version 1.0.0), DeepMicroFinder (‘hybrid’ model, commit a70f6d9), DeepVirFinder (version 1.0), PPR-Meta (version 1.1), Seeker (version 1.0.3), VIBRANT (version 1.2.1), viralVerify (version 1.1), VirSorter2 (version 2.2.3), Phigaro (version 2.3.0), PlasClass (version 0.1) and PlasX (commit 7349226). The tools were executed with default parameters and installed following the authors’ instructions, except for PPR-Meta, which was executed through a Docker container. VirSorter2 was also executed with the ‘--include-groups dsDNAphage,NCLDV,RNA,ssDNA,lavidaviridae’ parameter to measure its performance when using all classification models. To benchmark DeepMicroClass, we first assigned sequences to the class with the highest score. Next, we labeled the ones classified as ‘Eukaryote’ or ‘Prokaryote’ as chromosome and the ones assigned to ‘EukaryoteVirus’ or ‘ProkaryoteVirus’ as virus.

For the benchmarks that measured the sensitivity of virus detection across different viral and host taxa, we established cutoffs that approximated the FDR of each tool to 5%. The same was done in the benchmark that measured the sensitivity of plasmid detection across different host taxa, but we set the target FDR to 10%, as some tools could not achieve a 5% FDR regardless of the threshold. The procedure was performed to prevent overly sensitive tools (with elevated FDR) from dominating the benchmarks.

### Reporting summary

Further information on research design is available in the Nature Portfolio Reporting Summary linked to this article.

## Online content

Any methods, additional references, Nature Portfolio reporting summaries, source data, extended data, supplementary information, acknowledgements, peer review information; details of author contributions and competing interests; and statements of data and code availability are available at 10.1038/s41587-023-01953-y.

### Supplementary information

Supplementary Information Supplementary Notes 1–6 and Methods.

Reporting Summary

Supplementary Table Supplementary Tables 1–14.

## Data Availability

Metadata (specificity, functional annotation and hallmark information), multiple sequence alignments, HMMs and a MMseqs2 database of geNomad’s markers are available at 10.5281/zenodo.8303752. The taxonomically annotated viral protein database can be downloaded at 10.5281/zenodo.6574913. Reference sequences used for training and evaluation, the list of *P. aeruginosa* genomes used to build the pangenome and giant virus sequences discovered in metagenomes can be downloaded at 10.5281/zenodo.8049246. Sand Creek Marshes metatranscriptomes were retrieved from IMG/M (GOLD Study ID: Gs0142363).

## Code availability

geNomad is an open-source software, and its code can be found at https://github.com/apcamargo/genomad. The code used to build the taxonomically annotated viral protein database can be found at https://github.com/apcamargo/ictv-mmseqs2-protein-database. Python scripts used to train geNomad’s neural network and conditional random field models can be downloaded at 10.5281/zenodo.8049246.
