# Supplementary material for: Identification of mobile genetic elements with geNomad
Source: Nat Biotechnol. 2023 Sep 21;42(8):1303–12. doi: 10.1038/s41587-023-01953-y (PMC11324519; doi:10.1038/s41587-023-01953-y)
Supplement: Supplementary file 1 — Supplementary Notes 1–6 and Methods. [file 41587_2023_1953_MOESM1_ESM.pdf]

nature biotechnology

Article https://doi.org/10.1038/s41587-023-01953-y

Identification of mobile genetic elements with geNomad

In the format provided by the authors and unedited

Supplementary Information for "*Identification of mobile genetic elements with geNomad*"

Antonio Pedro Camargo[1,*], Simon Roux[1], Frederik Schulz[1], Michal Babinski[2], Yan Xu[2], Bin Hu[2], Patrick S. G. Chain[2], Stephen Nayfach[1], Nikos C. Kyrpides[1,*].

1. DOE Joint Genome Institute, Lawrence Berkeley National Laboratory, Berkeley, CA 94720, USA.

2. Bioscience Division, Los Alamos National Laboratory, Los Alamos, NM 87545, USA.

* Correspondence should be addressed to: antoniop.camargo@lbl.gov, nckyrpides@lbl.gov.

Supplementary Note 1: Marker-based classification features

To classify a given sequence into chromosome, plasmid, or virus with the marker-based classifier, geNomad performs gene prediction using prodigal-gv and annotates the predicted proteins by aligning them to geNomad's markers using MMseqs2. From the sequence's gene structure, RBS motifs, and the identity of the markers that were assigned to its proteins, a total of 25 informative features are computed and used as input for the classification model. Below we list and describe each one of these features:

- **`strand_switch_rate`:** The fraction of genes located on a different strand from the gene upstream.
- **`coding_density`:** Sum of the lengths of all the protein-coding regions (in base pairs) divided by the total sequence length.
- **`no_rbs_freq`:** Fraction of genes without a detectable RBS motif.
- **`sd_bacteroidetes_rbs_freq`:** Fraction of genes predicted to have a Bacteroidetes Shine-Dalgarno RBS motif (TAA, TAAA, TAAAA, TAAAT, TAAAAA, TAAAAT).
- **`sd_canonical_rbs_freq`:** Fraction of genes predicted to have a canonical Shine-Dalgarno RBS motif (3Base/5BMM, 4Base/6BMM, AGG, AGGA, AGGA/GGAG/GAGG, AGGAG, AGGAG/GGAGG, AGGAG(G)/GGAGG, AGGAGG, AGxAG, AGxAGG/AGGxGG, GAG, GAGG, GAGGA, GGA, GGA/GAG/AGG, GGAG, GGAG/GAGG, GGAGG, GGAGGA, GGxGG).
- **`tatata_rbs_freq`:** Fraction of genes predicted to have a TATATA RBS motif (ATA, ATAT, ATATA, ATATAT, TAT, TATA, TATAT, TATATA).
- **`cc_marker_freq`:** Number of genes assigned to the CC specificity class (high chromosome SPM, low plasmid SPM, low virus SPM) divided by the total number of genes.
- **`cp_marker_freq`:** Number of genes assigned to the CP specificity class (high chromosome SPM, medium plasmid SPM, low virus SPM) divided by the total number of genes.
- **`cv_marker_freq`:** Number of genes assigned to the CV specificity class (high chromosome SPM, low plasmid SPM, medium virus SPM) divided by the total number of genes.
- **`pc_marker_freq`:** Number of genes assigned to the PC specificity class (medium chromosome SPM, high plasmid SPM, low virus SPM) divided by the total number of genes.
- **`pp_marker_freq`:** Number of genes assigned to the PP specificity class (low chromosome SPM, high plasmid SPM, low virus SPM) divided by the total number of genes.
- **`pv_marker_freq`:** Number of genes assigned to the PV specificity class (low chromosome SPM, high plasmid SPM, medium virus SPM) divided by the total number of genes.
- **`vc_marker_freq`:** Number of genes assigned to the VC specificity class (medium chromosome SPM, low plasmid SPM, high virus SPM) divided by the total number of genes.

- **vp_marker_freq:** Number of genes assigned to the VP specificity class (low chromosome SPM, medium plasmid SPM, high virus SPM) divided by the total number of genes.
- **vv_marker_freq:** Number of genes assigned to the VV specificity class (low chromosome SPM, low plasmid SPM, high virus SPM) divided by the total number of genes.
- **c_marker_freq:** Total chromosome marker frequency (CC + CP + CV).
- **p_marker_freq:** Total plasmid marker frequency (PC + PP + PV).
- **v_marker_freq:** Total virus marker frequency (VC + VP + VV).
- **median_c_spm:** Median chromosome SPM across all annotated genes.
- **median_p_spm:** Median plasmid SPM across all annotated genes.
- **median_v_spm:** Median virus SPM across all annotated genes.
- **v_vs_c_score_logistic:** A sigmoid function is applied to a compound score($\sum_{i=1}^{n} \quad V\,SPM_i - C\,SPM_i$) to put it in the $[0-1]$ range.
- **v_vs_p_score_logistic:** A sigmoid function is applied to a compound score($\sum_{i=1}^{n} \quad V\,SPM_i - P\,SPM_i$) to put it in the $[0-1]$ range.
- **p_vs_v_score_logistic:** A sigmoid function is applied to a compound score($\sum_{i=1}^{n} \quad P\,SPM_i - V\,SPM_i$) to put it in the $[0-1]$ range.
- **gv_marker_freq:** Number of genes annotated with giant virus markers divided by the total number of genes.

Observations:

1. Predicted RBS motifs are extracted from prodigal-gv's gene prediction.
2. Each profile has three associated SPM values that range from 0 to 1 and measure how specific that profile is to each one of the three classes (chromosome, plasmid, and virus).
3. Markers were assigned to the nine specificity classes (CC, CP, CV, PC, PP, PV, VC, VP, and VV) based on their SPM values. Briefly, we used the "binned_statistic_dd" function from the SciPy Python library (version 1.7.3) to divide the three-dimensional SPM space into 125 equally sized bins. Next, each marker was assigned to a bin based on its SPM profile, so that all the markers within a given bin had similar chromosome, plasmid, and virus SPMs. Finally, we manually labeled each bin, and the markers within it, with the nine specificity classes, depending on their SPM profiles.
4. To label profiles as giant virus markers, we treated giant viruses (*Nucleocytoviricota*, *Pandoravirus*, *Mollivirus*, *Pithoviridae*, *Naldaviricetes*) as a fourth class, separate from all other viruses, and recomputed SPM values. Profiles with giant virus SPM ≥ 0.94 were considered giant virus markers. This threshold was picked based on the SPM of profiles of known *Megaviricetes* capsid proteins.

Supplementary Note 2: Score calibration

During the inference process, a classification model assigns scores to predictions, indicating the level of confidence in each prediction, where higher values signify greater confidence. However, these scores do not necessarily represent the true probabilities of the predictions being correct, as classification models will exhibit varying false discovery rates when classifying samples with distinct underlying compositions. For example, if the same classification model is used to identify viruses in a metagenome (where cellular sequences outnumber viral sequences) and in a virome (that is enriched in viral sequences), it is expected that the model will yield a higher proportion of false positive viruses in the metagenome, where more cellular sequences (that are prone to be misclassified as viruses) will be present (Extended Data Fig. 2A). This issue stems from the fact that models assign the same score to a given sequence regardless of the composition of the sample.

To address this, we devised an optional calibration mechanism in geNomad that leverages sample composition data to approximate the true underlying probabilities. The algorithm consists of a dense neural network that takes raw scores and the empirical sample composition (i.e., the frequency of chromosomes, plasmids, and viruses in the pre-calibration classification) as inputs and outputs calibrated scores (Fig. 1A, box A3) that accurately approximate probabilities (mean absolute errors for pre- and post-calibration scores in Fig. 1D). Because this process depends on reliable estimates of the underlying compositions, it works best for samples with sufficient size (e.g., ≥ 1,000 sequences), for which the mean absolute error of the calibration is very low (≈ 1%, Extended Data Fig. 2B). In essence, the calibration mechanism adjusts raw scores by reducing or increasing the scores of a given class (chromosome, plasmid, or virus) when its frequency within the sample is low or high (Extended Data Fig. 2C and D). When the sample composition is very uneven, this tends to result in large changes in raw scores, while very high or low scores are less affected (Extended Data Fig. 2C and E). The calibrated scores produced by geNomad offer users two benefits: (1) estimated probabilities can be used to compute false discovery rates, allowing users to make more informed decisions (e.g., setting a threshold to achieve a desired proportion of false positives), and (2) improved classification performance by adjusting the assigned labels of some sequences after calibrating scores.

Supplementary Note 3: Classification performance benchmarks

To evaluate the classification performance of geNomad and compare it to other virus and plasmid identification tools that use different approaches for sequence classification (Table 1), we used test datasets consisting of diverse sequence fragments with varying lengths (Extended Data Fig. 5A). To minimize overestimation of geNomad's performance due to the presence of similar sequences in the train and test data, we randomly assigned RCs to five different data splits and performed cross-validation using the leave-one-group-out strategy, which forced sequences from the same RC to remain together in either the train or test sets. Performance metrics for all tools were measured five times, using each RC as the test set at a time. The following metrics were computed: precision (fraction of true plasmids/viruses among the sequences classified as plasmid/virus); sensitivity (fraction of the true plasmids/viruses that were classified as such); Matthews correlation coefficient (MCC, correlation between the true and predicted labels); and F1-score (harmonic mean of sensitivity and precision).

geNomad exhibits better overall classification performance when compared to other tools

By inspecting the classification performance as a function of the similarity to the train data, we found that geNomad's performance dropped amongst sequences that were more divergent from the train data. However, it still performed rather well on unseen sequences (Extended Data Fig. 5B), especially viruses, illustrating its potential for the discovery of new viral taxa. Measurement of geNomad's performance on sequences with varying marker coverage (i.e., fraction of proteins assigned to markers) revealed that even those that were targeted by no or few markers were still detected due to the sequence branch of the algorithm (Extended Data Fig. 5C).

When compared to other tools, geNomad presented superior overall classification performance across all sequence length ranges in both plasmid and virus classification tasks (Fig. 3A and B, Supplementary Tables 3 and 4). The difference in performance was especially apparent in short sequences (< 6 kb): while the performance of most tools declined due to the limited genetic information in such sequences, geNomad leveraged its extensive marker dataset and alignment-free neural network to extract as much information as possible and maintain high sensitivity and precision. This highlights the usefulness of geNomad in metagenomic and metatranscriptomic assemblies, where most scaffolds are short.

Score calibration improves sequence classification

geNomad's calibration mechanism enhances the classification process by incorporating sample composition data and assigning estimated probabilities to each sequence, which reflect the likelihood of the sequence belonging to each class. By using calibrated scores instead of raw scores to assign labels, the average classification performance improves because biases introduced during model training are corrected. Indeed, our analysis showed that the plasmid classification performance increased with the use of calibrated scores, particularly for shorter sequences (average ΔMCC: +11.8% for sequences < 3 kb, +5.6% for 3–6 kb, and +3.2% for 6–9 kb) (Extended Data Fig. 5D). We also found that short virus sequences

benefited from calibration, though the improvement was not as pronounced. These results showcase the effectiveness of the introduced calibration mechanism for improving classification quality.

Plasmid classification benchmarks

Plasmid classification is a challenging task due to the variable genetic makeup of these elements, their similarity to other mobile elements that can integrate into host chromosomes, and the lack of a standard for reporting plasmids in sequencing data. As a result, most evaluated tools (DeepMicroClass[1], PPR-Meta[2], PlasClass[3], and viralVerify[4]) had low average classification precision (11.0–40.1%, Supplementary Table 3), even when classifying long sequences (Supplementary Table 4), as they often produced a high number of false positives that can impact downstream analysis. In contrast, PlasX[5] had high precision (81.6%), but low sensitivity (40.5%), which impairs the detection of plasmids in sequencing data. geNomad had the best overall performance by a substantial margin (Fig. 3A, MCC and F1-score in Supplementary Tables 3 and 4), with the highest sensitivity (89.8%) and the second highest precision (70.8%), after PlasX. It's worth noting that geNomad's marker branch, which can be run independently, achieved a considerably higher precision than PlasX (91.2%).

Most of the plasmid sequences in public databases are limited to a few taxa, such as *Gammaproteobacteria* and *Bacilli*, which can bias the training process if taxonomic imbalance is not taken into account. Because it was designed to reduce the effects of taxonomic representation biases during marker selection and training, geNomad is able to identify plasmids from underrepresented groups more accurately. A similar process was also used in PlasX. When compared to other plasmid identification tools, geNomad had the best performance across all appraised taxa (Supplementary Table 5). Notably, geNomad was the only tool to accurately identify the majority of *Archaea* plasmids (92.54%), which were frequently missed by other tools (0.0–55.3%), and it greatly outperformed other tools for identifying plasmids from major phyla such as *Cyanobacteria* (geNomad: 96.7%, other tools: 6.3–64.3%), *Actinobacteria* (geNomad: 95.5%, other tools: 2.5–61.9%), and *Bacteroidota* (geNomad: 86.4%, other tools: 2.4–69.2%).

Plasmid identification algorithms can be affected by low quality plasmid annotations in public data. Extrachromosomal viruses and secondary chromosomes are often incorrectly labeled as plasmids in these databases, so it's important to carefully filter the data to train reliable models and assess classification performance (details in the *Methods* section). To evaluate if existing plasmid identification tools are prone to misclassifying viruses as plasmids — possibly due to contamination in the training data — we measured the fraction of viruses in our test dataset that were labeled as plasmids by the benchmarked tools (Supplementary Table 6). geNomad, PlasX, and viralVerify had the best performances in this benchmark (1.7%, 1.5%, and 3.7% respectively), while DeepMicroClass, PlasClass, and PPR-Meta performed the worst (11.3%, 64.4%, and 9.8% respectively). Of note, geNomad's marker branch classified only 0.2% of the virus sequences as plasmids, which highlights the limitations of current alignment-free tools at this task and the importance of careful dataset curation.

Virus classification benchmarks

In virus classification, geNomad attained the best overall performance when considering all length strata (MCC: 95.3%, F1-score: 97.3%), followed by VirSorter2[6] executed with all models (MCC: 81.3%, F1-score: 88.9%), VirSorter2 executed with default parameters (MCC: 79.7%, F1-score: 87.1%), and PPR-Meta (MCC: 77.4%, F1-score: 86.6%) (Fig. 3B, Supplementary Table 3). VIBRANT[7], geNomad, VirSorter2 (default parameters), and DeepMicroClass achieved the highest classification precision (97.5%, 97.3%, 94.7%, and 92.6%, respectively), while Seeker[8], DeepVirFinder[9], and PPR-Meta obtained the lowest scores (61.8%, 80.5%, and 88.5%, respectively). VIBRANT's overall classification performance metrics appeared low (MCC: 36.0%, F1-score: 35.2%) due to its very low sensitivity when classifying short sequences (Fig. 3B, Supplementary Table 4), a consequence of it not classifying sequences that encode less than four genes and not being designed to identify eukaryotic viruses (see paragraph below).

The development of tools that can accurately identify diverse viral taxa is challenging, as no genes are universally shared across the virosphere. Additionally, unequal representation of viral groups — illustrated by the dominance of tailed phages from the *Caudiviricetes* class — in sequencing data can bias classification models and prevent the discovery of underrepresented taxa. In a benchmark study using representative genomes from the ICTV, we found that geNomad outperformed other tools in all major taxa we evaluated (Fig. 3C, Supplementary Table 7). Notably, geNomad was the only tool that achieved high sensitivity for viruses that encode an RNA-dependent RNA polymerase (*Orthornavirae*, 98.64%), and giant viruses (*Megaviricetes*, 94.74%) at a fixed false discovery rate of 5%. The only other tools to display sensitivity over 50% for all taxa were DeepMicroClass and viralVerify, while the remaining tools failed to achieve this for at least two of the groups. When evaluating sensitivity across different host clades, we found that geNomad was the only tool that identified more than 90% of the viruses infecting bacteria, archaea, and multiple eukaryotic groups, while other tools struggled to identify viruses that infect at least two of the eukaryotic groups that were evaluated (Supplementary Table 8). In an additional benchmark where we measured classification sensitivity on a catalog of metagenomic *Inovirus*[10], which are known to be challenging to detect automatically, geNomad (sensitivity: 84.8%) also outperformed other evaluated tools (average sensitivity: 32.5%, Supplementary Table 9). These results show that geNomad can be employed to identify a wide range of virus taxa infecting a variety of hosts, enabling the discovery of viruses that are often missed in metagenomic analyses, such as non-tailed phages and viruses that infect eukaryotes. It is worth noting that several of the tested tools (DeepVirFinder, PPR-Meta, Seeker, and VIBRANT) were trained only on phage data and are therefore not designed to identify viruses that don't infect prokaryotes. In fact, VIBRANT was a top performer for *Caudoviricetes*, *Tokiviricetes*, *Tubulavirales*, and *Microviridae*.

Supplementary Note 4: Evaluation of provirus detection in the *Pseudomonas aeruginosa* pangenome

We conducted a comparative genomics analysis to evaluate the performance of geNomad in predicting proviruses, in comparison to other provirus prediction tools. We employed PPanGGOLiN[11] (version 1.2.74) to create a *Pseudomonas aeruginosa* pangenome from 442 genomes and to identify its core genes, which are persistent across genomes and are not expected to be found within proviruses. Next, we measured the fraction of core genes in each predicted provirus region as a proxy for contamination and found that, compared to the other evaluated tools, geNomad retrieved more proviruses that tended to have low contamination levels (Extended Data Fig. 6A, Supplementary Table 11). To illustrate the importance of precise boundary demarcation for downstream biological interpretation, we show that geNomad was able to find provirus-encoded defense systems — such as DarTG[12] and Hachiman[13], detected with DefenseFinder[14] (version 1.0.9) — that were missed by overly conservative tools (Phigaro and VIBRANT) while excluding core host genes that were left within prophages by VirSorter2. DarTG was found right next to an integrase, illustrating how leveraging tRNAs and integrases for boundary prediction can improve interpretation of the phage-host interactions (Extended Data Fig. 6B).

Supplementary Note 5: geNomad enables accurate identification of RNA viruses in metatranscriptomic data

Recent studies have revealed a previously undetected diversity of RNA viruses from the *Orthornavirae* kingdom by performing large-scale metatranscriptome surveys[15–17]. However, these surveys are limited by their reliance on detecting the RNA-dependent RNA polymerase (RdRP) hallmark gene, thus systematically overlooking genome segments that do not encode RdRP and fragmented scaffolds missing this gene. As geNomad leverages an extensive set of markers covering diverse functions (1,293 out of the 1,906 markers assigned to *Orthornavirae* are not functionally annotated as RdRP) and an alignment-free classification model that doesn't rely on gene families, we tested whether it could reliably detect segments or fragmented sequences of RNA viruses that are missing the RdRP gene. To evaluate this, we gathered likely RNA virus sequences that do not encode RdRP by binning metatranscriptomes from microbial communities in the Sand Creek Marshes[18] based on high read coverage correlation with RdRP-encoding scaffolds. The co-occurrence of a given sequence with another encoding the hallmark protein across multiple samples suggests that they came from the same *Orthornavirae* genome. This binning-based approach does not rely on features used by geNomad for classification to identify those scaffolds, allowing us to avoid potential biases in our analysis.

In total, we identified 623 scaffolds that co-occurred with RdRP-encoding sequences across 34 metatranscriptome assemblies. The majority of these scaffolds (98.1%) were classified as viruses, indicating that geNomad is capable of identifying sequences of RNA virus genomes even when they lack the RdRP hallmark gene (Fig. 5A). When evaluating how other tools classify these sequences we found that, on average, only 43.7% of the scaffolds were classified as viral and that alignment-free models presented a higher sensitivity (Supplementary Table 12), highlighting that such scaffolds are often not targeted by markers. As expected, sequences containing RdRP genes were almost always classified as viral (99.9%, Fig. 5A). Inspection of pairs of co-occurring scaffolds revealed that they fell into two categories: (1) linear genomes that were assembled into two sequence fragments, one of which lacked the RdRP gene (*Marnaviridae* bin in Fig. 5B); and (2) segmented genomes, where the genome is encoded across multiple DNA molecules, only one of which encodes the RdRP (*Cystoviridae* bin in Fig. 5B). Closer examination of these sequences revealed that they encoded domains associated with viral function, such as helicases, proteases, and structural proteins. Many of these domains were covered by geNomad's markers (coloured genes in Fig. 5B), demonstrating that the use of an extensive set of protein profiles enabled geNomad to sensitively identify fragments of RNA virus genomes. Among sequences not encoding RdRP and not binned with RdRP-encoding scaffolds, yet classified as viruses by geNomad, we found fragments of RNA virus genomes missing the RdRP gene (*Leviviridae* scaffold in Fig. 5B) and transcripts of DNA viruses (*Caudoviricetes* scaffold in Fig. 5B).

Supplementary Note 6: Expanding the giant virus diversity through the application of geNomad in metagenomic data

Giant viruses of the *Nucleocytoviricota* phylum possess large and complex genomes, and their virions can be as large as the cells of many bacteria and archaea[19]. Due to their expansive genomes and diverse genetic repertoires, the identification of these viruses through high-throughput methods is challenging and often relies on computationally expensive phylogenetic analyses and metagenomic binning, which limits the search space[20–22]. To make geNomad capable of sensitive detection of giant viruses in sequencing data, we expanded the diversity of *Nucleocytoviricota* in the training data by including genomes identified in a previous metagenomic survey (Schulz *et al.* 2020)[22]. Additionally, we included classification features specifically designed to enhance their detection, such as frequency of giant virus-specific markers and the TATATA motifs (Supplementary Table 1, Supplementary Note 1). As a result, we found that geNomad outperformed other tools in the classification of *Megaviricetes* giant viruses (Fig. 3C).

To assess geNomad's capability to uncover new clades of giant viruses in sequencing data, we applied it to 28,865 metagenome assemblies from the IMG/M[23] database. Scaffolds classified as virus by geNomad that were at least 50 kb in length were further analyzed using the GVClass pipeline, which placed *Nucleocytoviricota* scaffolds in a phylogenetic context by identifying a set of conserved protein families and reconstructing gene trees together with reference genomes. A total of 11,414 scaffolds identified by geNomad were phylogenetically placed in the *Nucleocytoviricota* tree (median length: 73.3 kb, interquartile range: 58.6–102.7 kb, Fig. 5C, Supplementary Table 13). Other tools classified, on average, 77.4% of these scaffolds as viral (Supplementary Table 14). To compare the results with those obtained using the pipeline described in Schulz *et al.* (2020), we examined metagenomes that were processed using both methodologies and found that 1,562 sequences (43% of total) were only detected by geNomad, 1,976 scaffolds (55%) were identified by both methodologies, and only 74 (2%) were found exclusively in the previous survey, demonstrating that geNomad allowed increased recovery of *Nucleocytoviricota* sequences.

The majority of the giant virus sequences identified by geNomad were found to belong to the *Mesomimiviridae* family (n = 6,372) of the *Imitervirales* order (n = 8,915), which includes viruses of haptophytes and ochrophytes[24] (Fig. 5C, Supplementary Table 13). By measuring the increase in phylogenetic diversity brought by scaffolds from this survey, we found that the diversity of multiple orders was substantially expanded (Fig. 5C, Supplementary Table 13), particularly that of *Asfuvirales* (2.7× increase) and *Algavirales* (2.3× increase). Within metagenomes from soils, an understudied niche for giant viruses[25], we identified 235 additional *Nucleocytoviricota* scaffolds, up from 16 metagenomic bins reported in the previous survey. Phylogenetic reconstruction of these soil giant viruses revealed that they include several novel clades of *Imitervirales*, *Pimascovirales*, and *Asfuvirales* that do not have representatives in GenBank or Schulz *et al.* (2020) (Fig. 5D), suggesting that the underlying diversity of *Nucleocytoviricota* in soil is greatly underestimated.

Supplementary Methods

Ecosystem distribution of markers

The distribution of geNomad's markers across ecosystems was assessed by mapping the markers to proteins from public metagenomes and metatranscriptomes (retrieved from IMG/M on 2022-04-10) using MMseqs2[26] protein-profile search. The marker frequency matrix was then normalized using DESeq2[27] (version 1.34.0), by setting the size factor of each ecosystem (according to GOLD's ecosystem classification[28]) to the total number of proteins in it. Next, markers that mapped to less than 10 proteins were filtered out and DESeq2's variance stabilizing transformation was employed to transform the frequency matrix. To generate the RadViz visualizations from the transformed matrix, the Radviz R library (version 0.9.3) was used.

Training of the IGLOO-based alignment-free classification model

The sequence-based classifier was trained using a two-step supervised contrastive learning approach[29] (Extended Data Fig. 1). In the first step we trained an IGLOO[30] encoder to learn to produce vector representations of nucleotide sequences in such a way that sequences of the same class will tend to be clustered together and separate from sequences of different classes. To achieve this, input sequences are converted into 4-mer vectors (step size = 1) that are one-hot-encoded and zero-padded to 5,997 elements, which correspond to the number of 4-mers in a 6 kb sequence. These inputs are then fed to an IGLOO encoder, trained using the supervised contrastive loss, that produces 512-dimensional embeddings where sequence representations from the same class (chromosome, plasmid, or virus) are brought closer together, while maintaining a greater distance from sequence representations of different classes. The IGLOO encoder begins processing one-hot-encoded matrices by applying 128 convolutional filters to generate sequence feature maps. To gather relationships between non-contiguous parts of the sequence, IGLOO generates 2,100 patches, each containing slices extracted from random positions within the sequence. These patches are subsequently integrated in a self-attention mechanism, where different parts of the feature map are weighted, leveraging the long-range dependencies encoded in the patches, to derive the final sequence representation. In the second step, we trained a dense neural network classifier on top of the IGLOO representations using a focal loss[31], which forces the model to focus on hard-to-classify sequences. For inference, sequences longer than 5,997 bp are split into multiple non-overlapping windows whose scores are averaged at the end of the classification. To account for class imbalance and taxonomic bias during the training of both the encoder and classifier models, sequences were weighted in accordance with their RC. For both models, training was conducted using the Adam optimizer with gradient centralization[32]. Hyperparameter tuning (k-mer length used for sequence encoding, number of IGLOO patches, number of filters in IGLOO's convolutional layers, size of the filters, dimensionality of the classifier hidden layer) was performed with KerasTuner (version 1.1.0) using the HyperBand algorithm[33].

Binning of the Sand Creek Marshes metatranscriptomes

To evaluate whether geNomad is able to identify RNA virus segments that don't encode the RdRP hallmark gene, we retrieved the raw sequencing data and the assemblies of 34 metatranscriptome samples from microbial communities from the Sand Creek Marshes (GOLD Study ID: Gs0142363)[18] from IMG/M. Scaffolds shorter than 2 kb were discarded and the remaining sequences were classified using geNomad. Scaffolds encoding RdRP were identified by performing protein prediction with prodigal-gv and using the predicted proteins as queries to search against a database of RdRP HMM models[17] using hmmsearch[34] (parameter: "-E 1e-5"). Using minimap2[35] (version 2.24, parameters: "-N 5 -ax sr"), sequencing reads from each sample were independently mapped to sequences in a combined assembly, which was generated by concatenating the assemblies from individual samples. Then, we used samtools[36] (version 1.16.1) to sort the mapped reads and input them into CoverM (version 0.6.1, parameter: "-m metabat", available at https://github.com/wwood/CoverM), which measured scaffold coverage across samples. To perform an initial binning of scaffolds based on co-abundance, we employed Vamb[37] (version 3.0.2, parameter: "-a 0.025"). Bins containing RdRP-encoding sequences were refined by retaining only the scaffolds that presented high correlation to the coverage of the RdRP-encoding scaffold (Pearson correlation coefficient ≥ 0.95). To prevent spurious correlations, we only considered RdRP-encoding sequences with high prevalence (coverage > 0 in at least 20% of the samples).

Metagenomic survey and phylogenetic analysis of giant viruses

From a set of 28,865 metagenomes (retrieved on 2022-04-10 from IMG/M) we selected scaffolds longer than 50 kb that were classified as viruses by geNomad and subjected them to further processing using GVClass (version 0.9.3, available at https://github.com/NeLLi-team/gvclass/), a framework that identifies giant viruses and assigns them to taxonomic lineages using a phylogenetic placement approach. Briefly, we identified nine conserved giant virus orthologous groups (GVOGs)[38] using hmmsearch and used these GVOGs as queries for DIAMOND[39] searches against databases of the respective GVOGs, which were built from a representative set of bacteria, archaea, eukaryotes, and viruses. We extracted the top 100 hits, combined them with the query sequences, and aligned them with MAFFT[40] (version 7.490). The alignments were then trimmed with trimAl[41] (version 1.4, parameter: "-gt 0.1") and used to build a phylogenetic tree with FastTree[42] (version 2.1.11, parameters: "-spr 4 -mlacc 2 -slownni -lg"). To determine the final classification, we identified the nearest neighbor in the tree using branch lengths and the existing taxonomic string for that reference genome. The taxonomic strings from all identified nearest neighbors were then compared at different taxonomic ranks (genus, family, order, class, phylum) to yield the final classification at the lowest taxonomic rank on which all nearest neighbors agreed.

To measure the phylogenetic diversity (PD) gained by identifying giant viruses with geNomad, we extracted DNA PolB orthologs encoded by these sequences and by genomes from two external sources (Schulz et al., 2020, Aylward et al., 2021; only sequences on scaffolds longer than 50 kb were considered) using the DNA PolB HMM model from the GVOG database (GVOGm0054). These protein sequences were

aligned with MAFFT, trimmed with trimAl, and used to build a phylogenetic tree with FastTree. We then performed separate alignments and built trees for each of the orders in the *Nucleocytoviricota*, with and without geNomad contigs. The increase in PD was then determined as the fold difference between the sum of the branch lengths for each viral order after adding the giant viruses identified with geNomad.

To build the phylogenetic tree that included the giant viruses identified from soil metagenomes using geNomad, we employed a representative set of giant viruses from Aylward et al. (2021) and added additional GVMAGs recovered from soil samples in Schulz et al. (2020). The sequences of the seven predominantly vertically inherited GVOGs were identified across all scaffolds using hmmsearch, aligned using MAFFT, and trimmed with trimA. Subsequently, a concatenated alignment was used as input to reconstruct a phylogenetic tree with IQ-TREE[43] (version 2.2.0.3, parameters: "-m LG+F+I+G4").

References

1. Hou, S., Cheng, S., Chen, T., Fuhrman, J. A. & Sun, F. DeepMicrobeFinder sorts metagenomes into prokaryotes, eukaryotes and viruses, with marine applications. *bioRxiv* (2021) doi:10.1101/2021.10.26.466018.
2. Fang, Z. *et al.* PPR-Meta: a tool for identifying phages and plasmids from metagenomic fragments using deep learning. *GigaScience* **8**, giz066 (2019).
3. Pellow, D., Mizrahi, I. & Shamir, R. PlasClass improves plasmid sequence classification. *PLOS Comput. Biol.* **16**, e1007781 (2020).
4. Antipov, D., Raiko, M., Lapidus, A. & Pevzner, P. A. MetaviralSPAdes: assembly of viruses from metagenomic data. *Bioinformatics* **36**, 4126–4129 (2020).
5. Yu, M. K., Fogarty, E. C. & Eren, A. M. The genetic and ecological landscape of plasmids in the human gut. *bioRxiv* (2020) doi:10.1101/2020.11.01.361691.
6. Guo, J. *et al.* VirSorter2: a multi-classifier, expert-guided approach to detect diverse DNA and RNA viruses. *Microbiome* **9**, 37 (2021).
7. Kieft, K., Zhou, Z. & Anantharaman, K. VIBRANT: automated recovery, annotation and curation of microbial viruses, and evaluation of viral community function from genomic sequences. *Microbiome* **8**, 90 (2020).
8. Auslander, N., Gussow, A. B., Benler, S., Wolf, Y. I. & Koonin, E. V. Seeker: alignment-free identification of bacteriophage genomes by deep learning. *Nucleic Acids Res.* **48**, e121–e121 (2020).
9. Ren, J. *et al.* Identifying viruses from metagenomic data using deep learning. *Quant. Biol.* **8**, 64–77 (2020).
10. Roux, S. *et al.* Cryptic inoviruses revealed as pervasive in bacteria and archaea across Earth's biomes. *Nat. Microbiol.* **4**, 1895–1906 (2019).

11. Gautreau, G. *et al.* PPanGGOLiN: Depicting microbial diversity via a partitioned pangenome graph. *PLOS Comput. Biol.* **16**, e1007732 (2020).

12. LeRoux, M. *et al.* The DarTG toxin-antitoxin system provides phage defence by ADP-ribosylating viral DNA. *Nat. Microbiol.* **7**, 1028–1040 (2022).

13. Doron, S. *et al.* Systematic discovery of antiphage defense systems in the microbial pangenome. *Science* **359**, eaar4120 (2018).

14. Tesson, F. *et al.* Systematic and quantitative view of the antiviral arsenal of prokaryotes. *Nat. Commun.* **13**, 2561 (2022).

15. Edgar, R. C. *et al.* Petabase-scale sequence alignment catalyses viral discovery. *Nature* **602**, 142–147 (2022).

16. Zayed, A. A. *et al.* Cryptic and abundant marine viruses at the evolutionary origins of Earth's RNA virome. *Science* **376**, 156–162 (2022).

17. Neri, U. *et al.* Expansion of the global RNA virome reveals diverse clades of bacteriophages. *Cell* **185**, 4023-4037.e18 (2022).

18. H. Vineis, J. Nutrient influence on microbial structure and function within salt marsh sediments. (Northeastern University, 2022).

19. Koonin, E. V. & Yutin, N. Origin and Evolution of Eukaryotic Large Nucleo-Cytoplasmic DNA Viruses. *Intervirology* **53**, 284–292 (2010).

20. Schulz, F. *et al.* Giant viruses with an expanded complement of translation system components. *Science* **356**, 82–85 (2017).

21. Bäckström, D. *et al.* Virus Genomes from Deep Sea Sediments Expand the Ocean Megavirome and Support Independent Origins of Viral Gigantism. *mBio* **10**, e02497-18 (2019).

22. Schulz, F. *et al.* Giant virus diversity and host interactions through global metagenomics. *Nature* **578**, 432–436 (2020).

23. Chen, I.-M. A. *et al.* The IMG/M data management and analysis system v.7: content updates and new features. *Nucleic Acids Res.* **51**, D723–D732 (2023).

24. Gallot-Lavallée, L., Blanc, G. & Claverie, J.-M. Comparative Genomics of Chrysochromulina Ericina Virus and Other Microalga-Infecting Large DNA Viruses Highlights Their Intricate Evolutionary Relationship with the Established Mimiviridae Family. *J. Virol.* **91**, e00230-17 (2017).

25. Schulz, F. *et al.* Hidden diversity of soil giant viruses. *Nat. Commun.* **9**, 4881 (2018).

26. Steinegger, M. & Söding, J. MMseqs2 enables sensitive protein sequence searching for the analysis of massive data sets. *Nat. Biotechnol.* **35**, 1026–1028 (2017).

27. Love, M. I., Huber, W. & Anders, S. Moderated estimation of fold change and dispersion for RNA-seq data with DESeq2. *Genome Biol.* **15**, 550 (2014).

28. Mukherjee, S. *et al.* Twenty-five years of Genomes OnLine Database (GOLD): data updates and new features in v.9. *Nucleic Acids Res.* **51**, D957–D963 (2023).

29. Khosla, P. *et al.* Supervised Contrastive Learning. in *Advances in Neural Information Processing Systems* (eds. Larochelle, H., Ranzato, M., Hadsell, R., Balcan, M. F. & Lin, H.) vol. 33 18661–18673 (Curran Associates, Inc., 2020).

30. Sourkov, V. IGLOO: Slicing the Features Space to Represent Sequences. *arXiv* (2018) doi:10.48550/ARXIV.1807.03402.

31. Lin, T.-Y., Goyal, P., Girshick, R., He, K. & Dollár, P. Focal Loss for Dense Object Detection. *IEEE Trans. Pattern Anal. Mach. Intell.* **42**, 318–327 (2020).

32. Yong, H., Huang, J., Hua, X. & Zhang, L. Gradient Centralization: A New Optimization Technique for Deep Neural Networks. in *Computer Vision – ECCV 2020* (eds. Vedaldi, A., Bischof, H., Brox, T. & Frahm, J.-M.) vol. 12346 635–652 (Springer International Publishing, 2020).

33. Li, L., Jamieson, K., DeSalvo, G., Rostamizadeh, A. & Talwalkar, A. Hyperband: A Novel Bandit-Based Approach to Hyperparameter Optimization. *J Mach Learn Res* **18**, 6765–6816 (2017).

34. Eddy, S. R. Accelerated Profile HMM Searches. *PLOS Comput. Biol.* **7**, e1002195 (2011).

35. Li, H. Minimap2: pairwise alignment for nucleotide sequences. *Bioinformatics* **34**, 3094–3100 (2018).

36. Li, H. *et al.* The Sequence Alignment/Map format and SAMtools. *Bioinformatics* **25**, 2078–2079 (2009).

37. Nissen, J. N. *et al.* Improved metagenome binning and assembly using deep variational autoencoders. *Nat. Biotechnol.* **39**, 555–560 (2021).

38. Aylward, F. O., Moniruzzaman, M., Ha, A. D. & Koonin, E. V. A phylogenomic framework for charting the diversity and evolution of giant viruses. *PLOS Biol.* **19**, e3001430 (2021).

39. Buchfink, B., Reuter, K. & Drost, H.-G. Sensitive protein alignments at tree-of-life scale using DIAMOND. *Nat. Methods* **18**, 366–368 (2021).

40. Katoh, K. & Standley, D. M. MAFFT Multiple Sequence Alignment Software Version 7: Improvements in Performance and Usability. *Mol. Biol. Evol.* **30**, 772–780 (2013).

41. Capella-Gutiérrez, S., Silla-Martínez, J. M. & Gabaldón, T. trimAl: a tool for automated alignment trimming in large-scale phylogenetic analyses. *Bioinformatics* **25**, 1972–1973 (2009).

42. Price, M. N., Dehal, P. S. & Arkin, A. P. FastTree 2 – Approximately Maximum-Likelihood Trees for Large Alignments. *PLOS ONE* **5**, e9490 (2010).

43. Minh, B. Q. *et al.* IQ-TREE 2: New Models and Efficient Methods for Phylogenetic Inference in the Genomic Era. *Mol. Biol. Evol.* **37**, 1530–1534 (2020).
