# Supplementary material for: Identification of mobile genetic elements with geNomad
Source: Nat Biotechnol. 2023 Sep 21;42(8):1303–12. doi: 10.1038/s41587-023-01953-y (PMC11324519; doi:10.1038/s41587-023-01953-y)
Supplement: Supplementary file 2 — Reporting Summary [file 41587_2023_1953_MOESM2_ESM.pdf]

nature portfolio

Corresponding author(s): Antonio Pedro Camargo

Last updated by author(s): Jun 19, 2023

Reporting Summary

Nature Portfolio wishes to improve the reproducibility of the work that we publish. This form provides structure for consistency and transparency in reporting. For further information on Nature Portfolio policies, see our Editorial Policies and the Editorial Policy Checklist.

Statistics

For all statistical analyses, confirm that the following items are present in the figure legend, table legend, main text, or Methods section.

n/a	Confirmed	
☐	☒	The exact sample size (*n*) for each experimental group/condition, given as a discrete number and unit of measurement
☐	☒	A statement on whether measurements were taken from distinct samples or whether the same sample was measured repeatedly
☒	☐	The statistical test(s) used AND whether they are one- or two-sided<br>*Only common tests should be described solely by name; describe more complex techniques in the Methods section.*
☒	☐	A description of all covariates tested
☒	☐	A description of any assumptions or corrections, such as tests of normality and adjustment for multiple comparisons
☐	☒	A full description of the statistical parameters including central tendency (e.g. means) or other basic estimates (e.g. regression coefficient) AND variation (e.g. standard deviation) or associated estimates of uncertainty (e.g. confidence intervals)
☒	☐	For null hypothesis testing, the test statistic (e.g. *F*, *t*, *r*) with confidence intervals, effect sizes, degrees of freedom and *P* value noted<br>*Give P values as exact values whenever suitable.*
☒	☐	For Bayesian analysis, information on the choice of priors and Markov chain Monte Carlo settings
☒	☐	For hierarchical and complex designs, identification of the appropriate level for tests and full reporting of outcomes
☒	☐	Estimates of effect sizes (e.g. Cohen's *d*, Pearson's *r*), indicating how they were calculated

Our web collection on statistics for biologists contains articles on many of the points above.

Software and code

Policy information about availability of computer code

Data collection

No software was used for data collection.

Data analysis

geNomad is an open-source software and its code can be found at https://github.com/apcamargo/genomad. The code used to build the taxonomically annotated viral protein database can be found at https://github.com/apcamargo/ictv-mmseqs2-protein-database. Python scripts used to train geNomad's neural network and conditional random field models can be downloaded from https://doi.org/10.5281/zenodo.8049246.

List of software: ARAGORN (version 1.2.41), BLAST (version 2.11.0+), CheckV (version 1.0.1), CoverM (version 0.6.1), DeepMicroClass (commit a70f6d9), DeepVirFinder (version 1.0), DefenseFinder (version 1.0.9), DESeq2 (version 1.34.0), DIAMOND (version 2.0.15), FastTree (version 2.1.11), geNomad (version 1.0.0), GVClass (version 0.9.3), HH-suite3 (version 3.3.0), HMMER (version 3.3.2), hypeR R library (version 1.13.0), 1Q-TREE (version 2.2.0.3), Kalign (version 3.3.1), KerasTuner (version 1.1.0), MAFFT (version 7.490), minimap2 (version 2.24), MMseqs2 (version 13-4511), Phigaro (version 2.3.0), PlasClass (version 0.1), PlasX (commit 7349226), PanGGOLiN (version 1.2.74), PPR-Meta (version 1.1), Prodigal (version 2.6.3), prodigal-gv (version 2.7.0), python-igraph (version 0.9.9), Radviz R library (version 0.9.3), samtools (version 1.16.1), Seeker (version 1.0.3), SetSimilaritySearch (version 0.1.7), shap-hypetune (version 0.2.4), TaxonKit (version 0.11.1), taxopy (version 0.9.2), trimAl (version 1.4), tspex (version 0.6.2), Vamb (version 3.0.2), VIBRANT (version 1.2.1), viralVerify (version 1.1), VirSorter2 (version 2.2.3), XGBoost (version 1.5.1).

For manuscripts utilizing custom algorithms or software that are central to the research but not yet described in published literature, software must be made available to editors and reviewers. We strongly encourage code deposition in a community repository (e.g. GitHub). See the Nature Portfolio guidelines for submitting code & software for further information.

Data

Policy information about availability of data

All manuscripts must include a data availability statement. This statement should provide the following information, where applicable:

- Accession codes, unique identifiers, or web links for publicly available datasets
- A description of any restrictions on data availability
- For clinical datasets or third party data, please ensure that the statement adheres to our policy

Metadata (specificity, functional annotation, hallmark information, etc.), multiple sequence alignments, HMMs, and a MMseqs2 database of geNomad's markers are available at https://doi.org/10.5281/zenodo.7586412. The taxonomically annotated viral protein database can be downloaded from https://doi.org/10.5281/zenodo.6574913. Reference sequences utilized for training and evaluation, the list of P. aeruginosa genomes used to build the pangenome, and giant virus sequences discovered in metagenomes can be downloaded from https://doi.org/10.5281/zenodo.8049246. Sand Creek Marshes metatranscriptomes were retrieved from IMG/M (GOLD Study ID: Gs0142363).

Human research participants

Policy information about studies involving human research participants and Sex and Gender in Research.

Reporting on sex and gender	n/a
Population characteristics	n/a
Recruitment	n/a
Ethics oversight	n/a

Note that full information on the approval of the study protocol must also be provided in the manuscript.

Field-specific reporting

Please select the one below that is the best fit for your research. If you are not sure, read the appropriate sections before making your selection.

☒ Life sciences ☐ Behavioural & social sciences ☐ Ecological, evolutionary & environmental sciences

For a reference copy of the document with all sections, see nature.com/documents/nr-reporting-summary-flat.pdf

Life sciences study design

All studies must disclose on these points even when the disclosure is negative.

Sample size	No calculation of the sample size was conducted since there was no statistical comparison performed between groups.
Data exclusions	No data were excluded.
Replication	No experimental replication was performed. Models were trained and evaluated using publicly available sequence data.
Randomization	To evaluate the model, clusters of similar sequences were randomly divided into five groups and used for cross-validation. The sequences were first clustered before splitting the dataset to minimize data leakage during the evaluation of the classification models.
Blinding	The investigators were not blinded to the datasets, as it would not have affected the analysis conducted in this study. The models were evaluated using test data that were kept separate from the training data.

Reporting for specific materials, systems and methods

We require information from authors about some types of materials, experimental systems and methods used in many studies. Here, indicate whether each material, system or method listed is relevant to your study. If you are not sure if a list item applies to your research, read the appropriate section before selecting a response.

Materials & experimental systems

n/a	Involved in the study
☒	☐ Antibodies
☒	☐ Eukaryotic cell lines
☒	☐ Palaeontology and archaeology
☒	☐ Animals and other organisms
☒	☐ Clinical data
☒	☐ Dual use research of concern

Methods

n/a	Involved in the study
☒	☐ ChIP-seq
☒	☐ Flow cytometry
☒	☐ MRI-based neuroimaging
